# Supplementary material for: Distinct Spatial Ca2+ Signatures Selectively Activate Different NFAT Transcription Factor Isoforms
Source: Mol Cell. 2015 Apr 16;58(2):232–43. doi: 10.1016/j.molcel.2015.02.027 (PMC4405353; doi:10.1016/j.molcel.2015.02.027)
Supplement: Document S1. Figures S1–S7 [file mmc1.pdf]

Molecular Cell

Supplemental Information

Distinct Spatial Ca^{2+} Signatures Selectively Activate Different NFAT Transcription Factor Isoforms

Pulak Kar and Anant B. Parekh

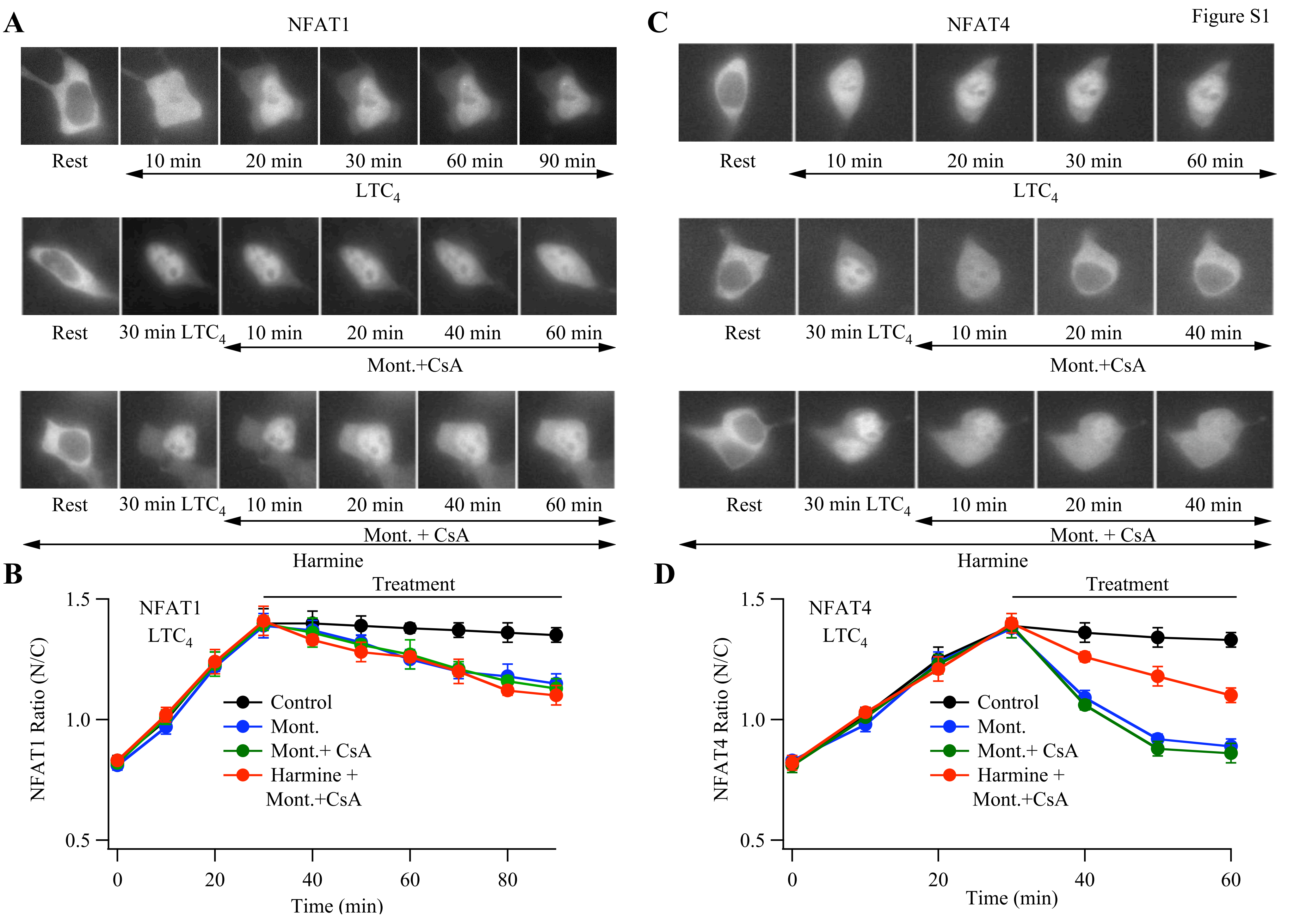

Figure S1
A
NFAT1
Rest
10 min
20 min
30 min
60 min
90 min
LTC4
Rest
30 min LTC4
10 min
20 min
40 min
60 min
Mont.+CsA
Rest
30 min LTC4
10 min
20 min
40 min
60 min
Mont. + CsA
Harmine
C
NFAT4
Rest
10 min
20 min
30 min
60 min
LTC4
Rest
30 min LTC4
10 min
20 min
40 min
Mont.+CsA
Rest
30 min LTC4
10 min
20 min
40 min
Mont. + CsA
Harmine
B
Treatment
NFAT1
LTC4
NFAT1 Ratio (N/C)
Time (min)
Control
Mont.
Mont.+ CsA
Harmine +
Mont.+CsA
D
Treatment
NFAT4
LTC4
NFAT4 Ratio (N/C)
Time (min)
Control
Mont.
Mont.+ CsA
Harmine +
Mont.+CsA

Figure S2

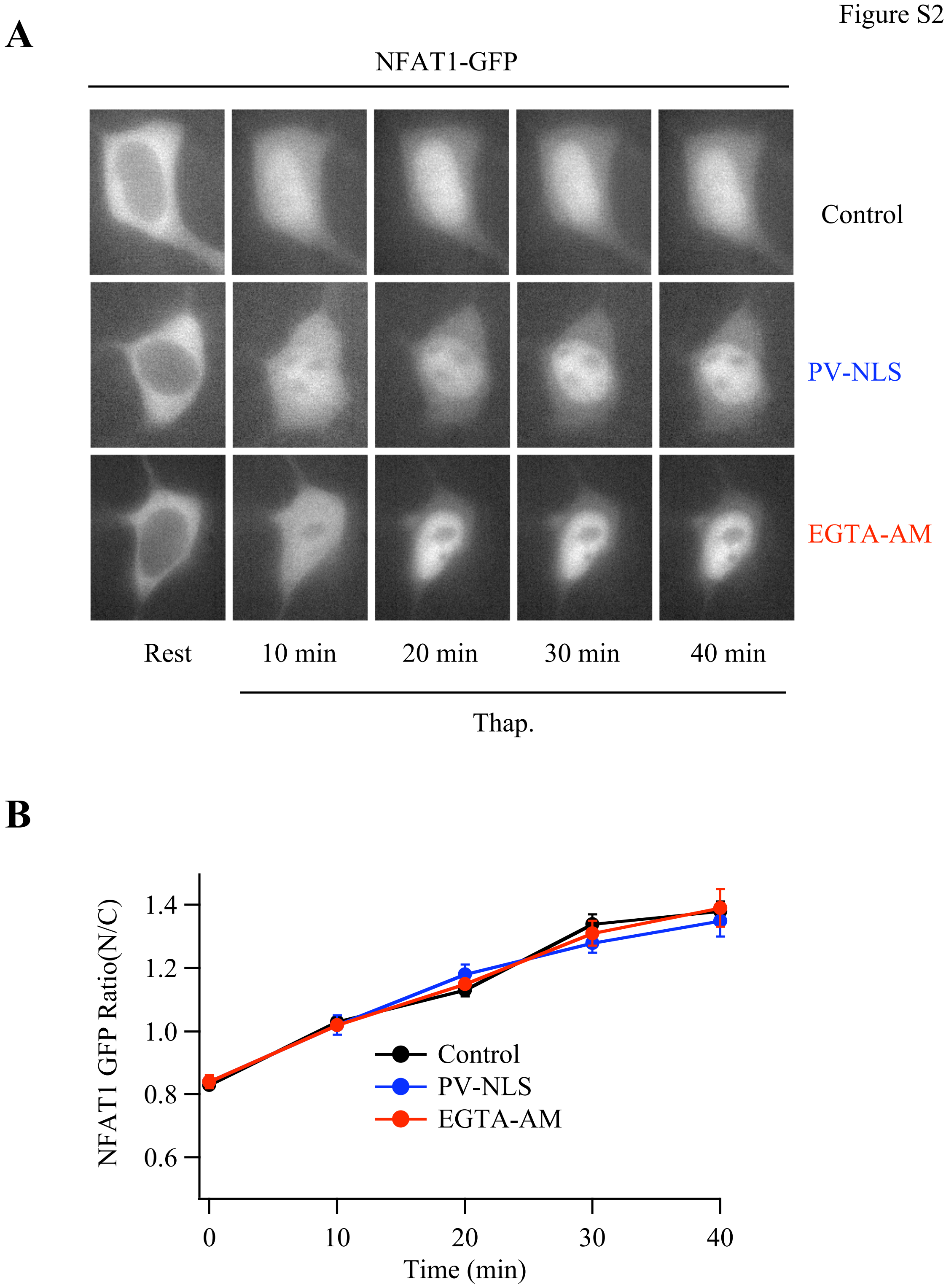
A
NFAT1-GFP
Control
PV-NLS
EGTA-AM
Rest
10 min
20 min
30 min
40 min
Thap.
B
NFAT1 GFP Ratio(N/C)
1.4
1.2
1.0
0.8
0.6
0
10
20
30
40
Time (min)
Control
PV-NLS
EGTA-AM

Figure S3

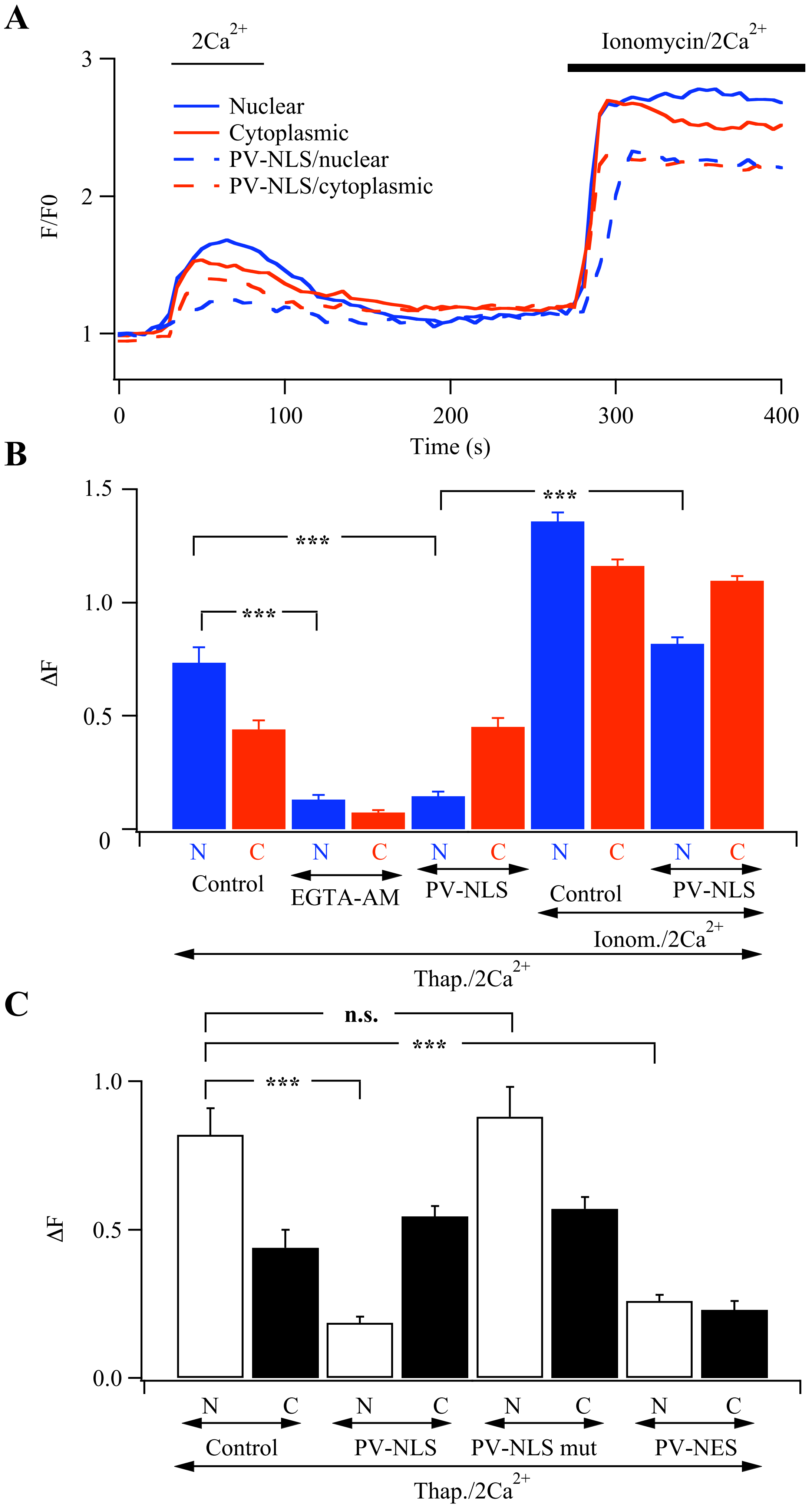
A
2Ca2+
Ionomycin/2Ca2+
Nuclear
Cytoplasmic
PV-NLS/nuclear
PV-NLS/cytoplasmic
F/F0
Time (s)
B
***
***
***
ΔF
N C N C N C N C N C
Control
EGTA-AM
PV-NLS
Control
PV-NLS
Ionom./2Ca2+
Thap./2Ca2+
C
n.s.
***
***
ΔF
N C N C N C N C
Control
PV-NLS
PV-NLS mut
PV-NES
Thap./2Ca2+

Figure S4

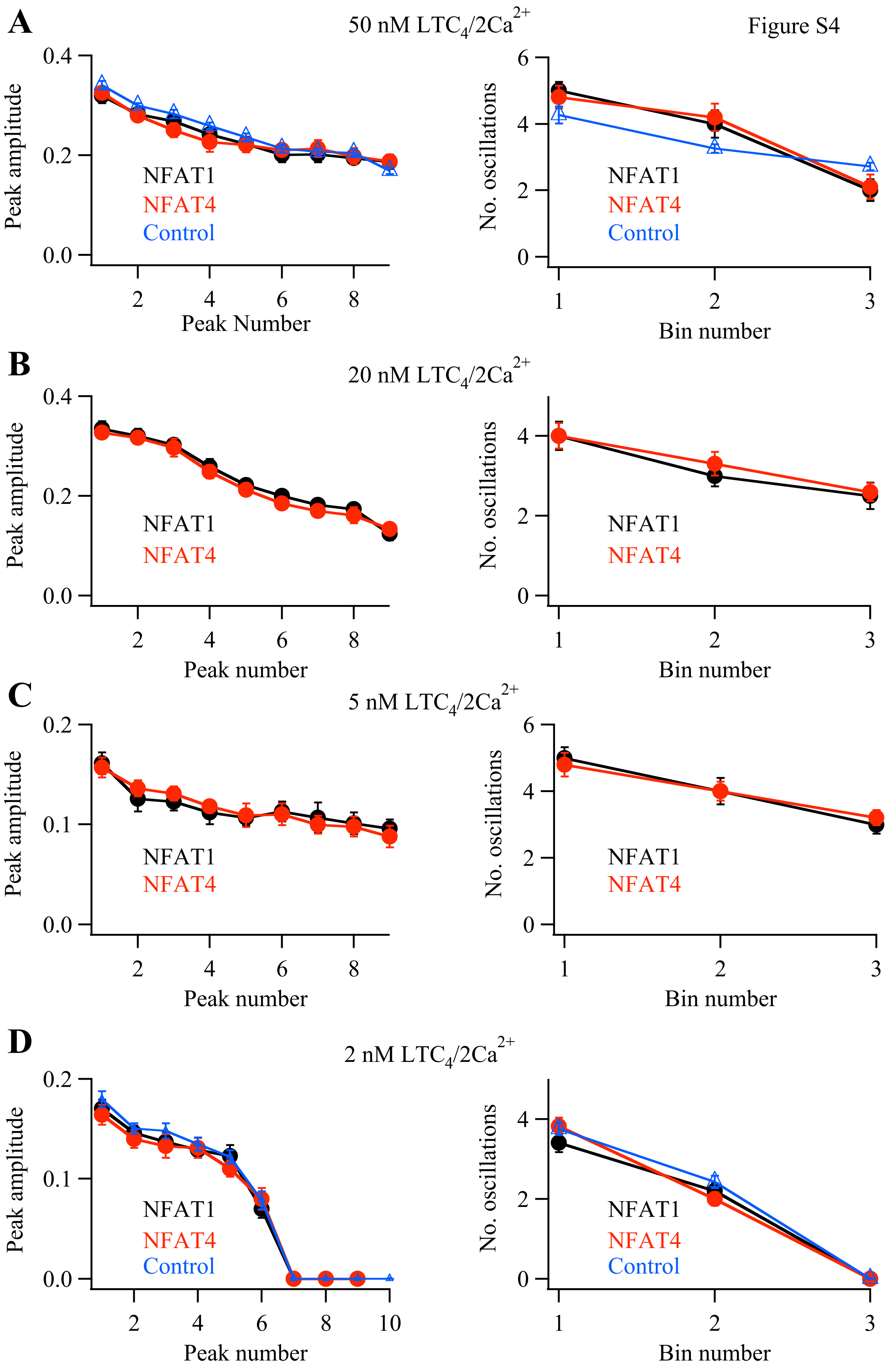

A
50 nM $LTC_4/2Ca^{2+}$
Peak amplitude
Peak Number
NFAT1
NFAT4
Control
No. oscillations
Bin number
NFAT1
NFAT4
Control
B
20 nM $LTC_4/2Ca^{2+}$
Peak amplitude
Peak number
NFAT1
NFAT4
No. oscillations
Bin number
NFAT1
NFAT4
C
5 nM $LTC_4/2Ca^{2+}$
Peak amplitude
Peak number
NFAT1
NFAT4
No. oscillations
Bin number
NFAT1
NFAT4
D
2 nM $LTC_4/2Ca^{2+}$
Peak amplitude
Peak number
NFAT1
NFAT4
Control
No. oscillations
Bin number
NFAT1
NFAT4
Control

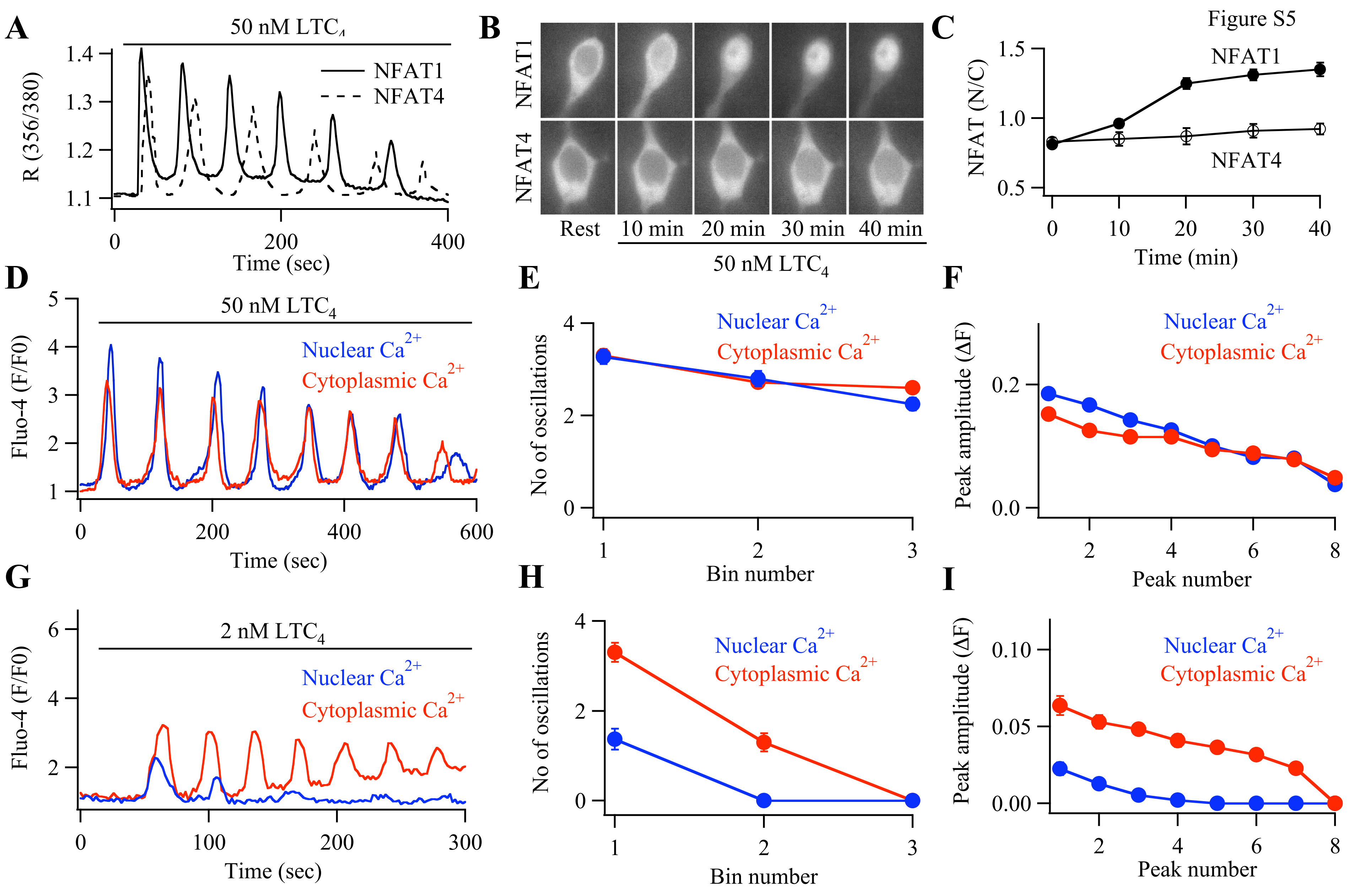

Figure S5
A
50 nM LTC4
NFAT1
NFAT4
R (356/380)
Time (sec)
B
NFAT1
NFAT4
Rest
10 min
20 min
30 min
40 min
50 nM LTC4
C
NFAT1
NFAT4
NFAT (N/C)
Time (min)
D
50 nM LTC4
Nuclear Ca2+
Cytoplasmic Ca2+
Fluo-4 (F/F0)
Time (sec)
E
Nuclear Ca2+
Cytoplasmic Ca2+
No of oscillations
Bin number
F
Nuclear Ca2+
Cytoplasmic Ca2+
Peak amplitude (ΔF)
Peak number
G
2 nM LTC4
Nuclear Ca2+
Cytoplasmic Ca2+
Fluo-4 (F/F0)
Time (sec)
H
Nuclear Ca2+
Cytoplasmic Ca2+
No of oscillations
Bin number
I
Nuclear Ca2+
Cytoplasmic Ca2+
Peak amplitude (ΔF)
Peak number

Figure S6

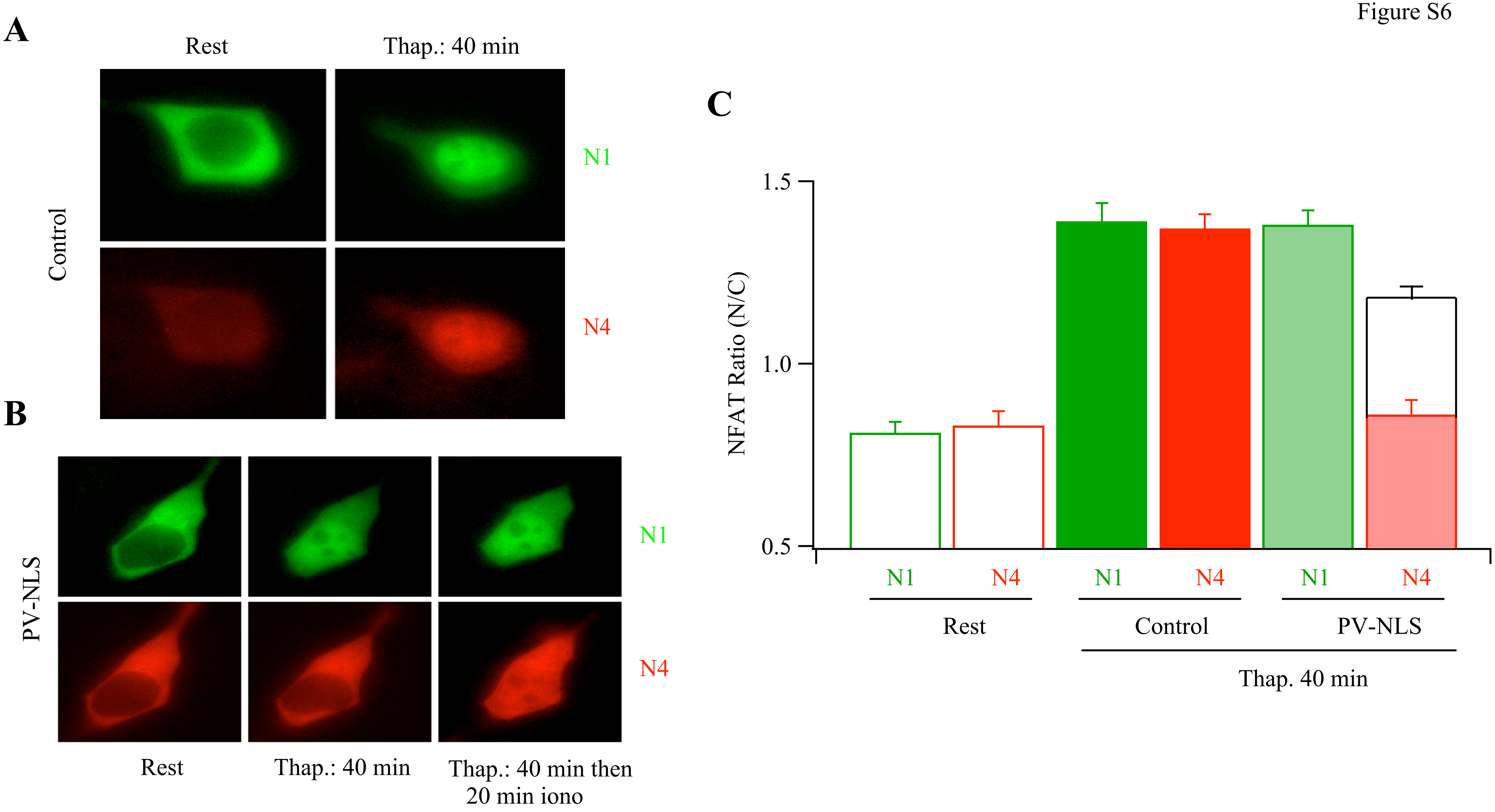
A
Rest
Thap.: 40 min
Control
N1
N4
B
PV-NLS
N1
N4
Rest
Thap.: 40 min
Thap.: 40 min then
20 min iono
C
NFAT Ratio (N/C)
1.5
1.0
0.5
N1
N4
N1
N4
N1
N4
Rest
Control
PV-NLS
Thap. 40 min

Figure S7

A

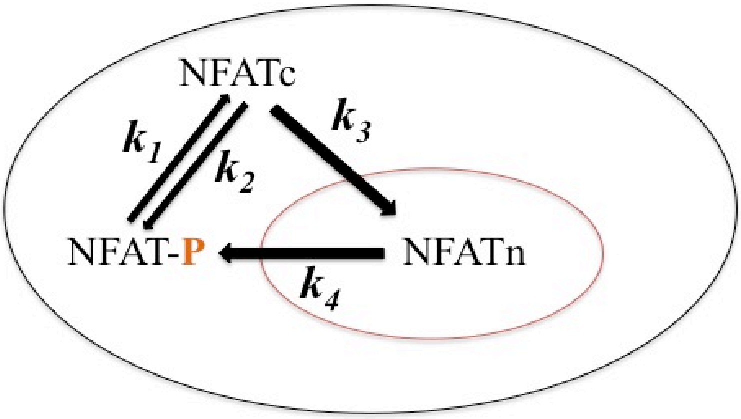

Cytoplasmic phosphorylated NFAT (NFAT-P) = X
Cytoplasmic dephosphorylated NFAT (NFATc) = Y
Nuclear NFAT (NFATn) = 1-X-Y

$$dx/dt = -k_1*X + k_2*Y + k_4(1\text{-}X\text{-}Y)$$
$$dy/dt = k_1*X-(k_2+k_3)Y$$

B

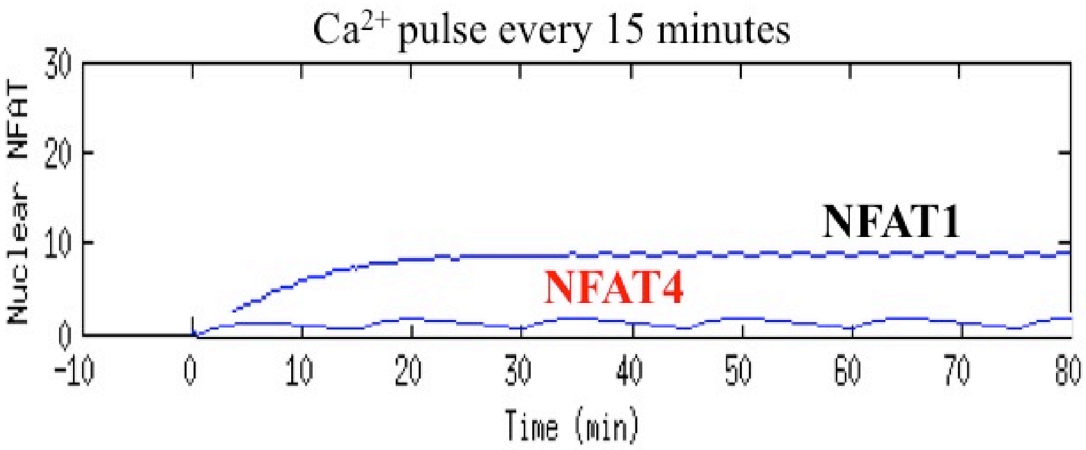

C

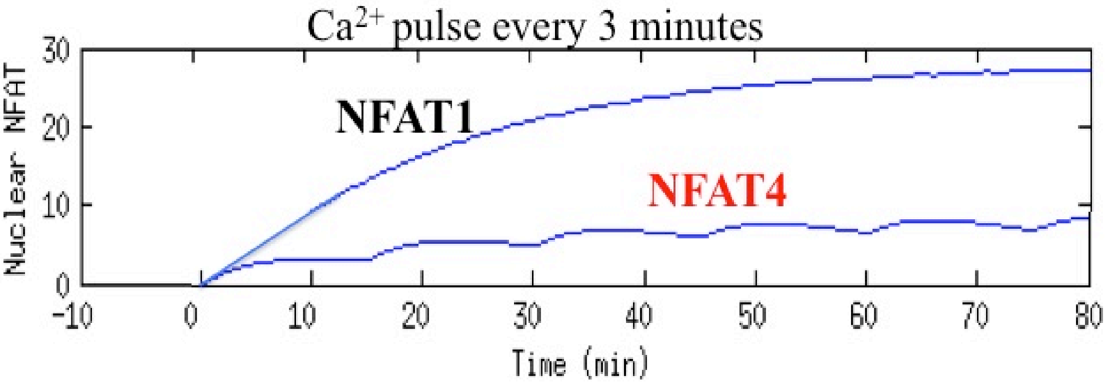

D

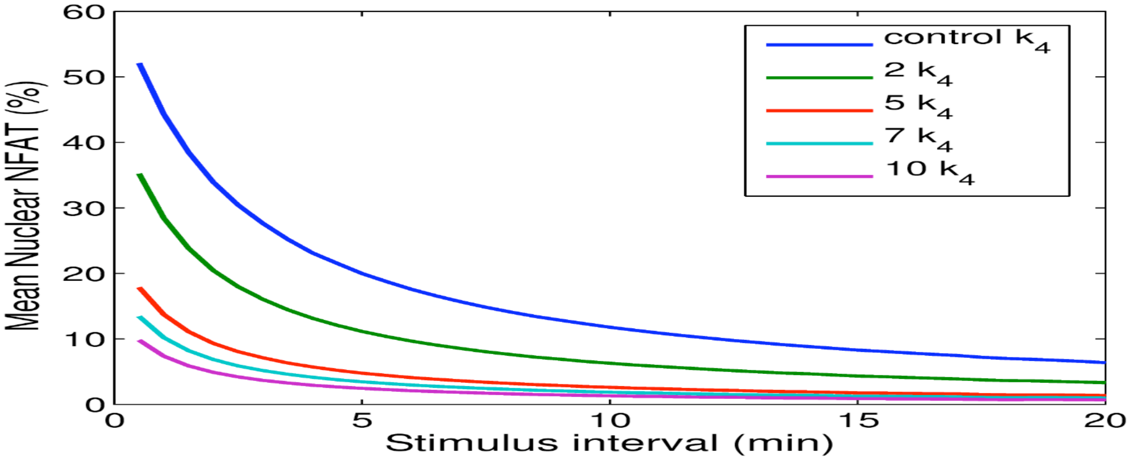

Figure S1. NFAT4 export from the nucleus is faster than that of NFAT1 following cysLT1 receptor stimulation, related to Figure 1. (A) Images compare NFAT1 nuclear dynamics following stimulation with 120 nM LTC_4. Upper panel shows NFAT1-GFP migration after exposure to agonist for the times indicated. Middle panel depicts NFAT1-GFP export from the nucleus following nuclear accumulation induced by stimulation with LTC_4 for 30 minutes. Thereafter, agonist was removed and cells exposed to montelukast (abbreviated to Mont.; 1 μM) and cyclosporine A (CsA; 1 μM). Images show NFAT1-GFP distribution at the times indicated. Lower panel, as in middle panel but now harmine was present too (5 μM pre-incubation for 10 minutes followed by continuous exposure). (B) Aggregate data for the conditions shown are compared. Each point is the average of between 11 and 19 cells. All cells were stimulated with LTC_4 shortly after resting images had been obtained. Control denotes the continuous presence of LTC_4, Mont. refers to addition of montelukast, Mont. + CsA represents montelukast and cyclosporine A and harmine + Mont.+CsA denotes the presence of all 3 inhibitors. (C) Images as in panel A but now NFAT4-GFP was expressed instead. (D) Aggregate data for NFAT4 for the various conditions are compared. Treatment was as described in panel B for NFAT1. Data are presented as mean±SEM.

Figure S2. Nuclear accumulation of NFAT1-GFP is unaffected by cytoplasmic Ca^{2+} buffering, related to Figure 3. (A) NFAT1-GFP translocation to the nucleus in response to thapsigargin stimulation is compared between a control cell, one expressing PV-NLS and one loaded with EGTA. (B) Mean data from several independent experiments are compared. Each point is the mean of between 6 and 13 cells. Error bars denote SEM.

Figure S3. Simultaneous measurements of nuclear and cytoplasmic Ca^{2+} signals, related to Figure 3. Nuclear and

cytoplasmic Ca^{2+} was measured with a confocal microscope using fluo-4. (A) Stores were depleted with thapsigargin in Ca^{2+}-free solution and 2 mM Ca^{2+} was readmitted, as indicated. Nuclear and cytoplasmic Ca^{2+} levels were measured in wild type cells and in cells expressing PV-NLS. PV-NLS/nuclear indicates nuclear Ca^{2+} in cells expressing PV-NLS. Ionomycin (2 μM) was applied after cytoplasmic Ca^{2+} had returned to basal levels. (B) Mean data for the conditions shown are presented as ΔF, denoting the Ca^{2+} response after stimulation minus the basal level. Each bar is the mean of between 6 and 13 cells. N and C denote nuclear and cytoplasmic Ca^{2+}, respectively. (C) Mean data for the conditions shown are compared. PV-NLS mut denotes the PV-NLS mutant that cannot bind Ca^{2+}. Error bars denote SEM. *** denotes $p<0.001$ and n.s. not significant.

Figure S4. Amplitude and frequency of cytoplasmic Ca^{2+} oscillations are maintained over a range of agonist concentrations in cells expressing cysLT1 receptors and NFAT proteins, related to Figure 4. (A) The amplitude of each oscillation and the number of oscillations per 200 seconds bin are compared following stimulation with 50 nM LTC_4. Control denotes cells expressing cysLT1 receptors alone, NFAT1 represents receptors and NFAT1 whereas NFAT4 denotes receptors and NFAT4. (B) Oscillatory responses are compared between cells expressing cysLT1 receptors and NFAT1 with cells expressing cysLT1 receptors and NFAT4 challenged with 20 nM LTC_4. (C) Oscillatory responses are compared as in panel B but now 5 nM LTC_4 was used. (D) Responses elicited by 2 nM LTC_4 are shown under the indicated conditions. Each point represents mean±SEM of 27-36 cells.

Figure S5. A nuclear Ca^{2+} rise is required for NFAT4 accumulation in the nucleus following physiological levels of stimulation, related to Figure 4. (A) A low dose of LTC_4 evokes

several Ca^{2+} oscillations of similar amplitude and frequency in cells expressing PV-NLS (untagged) and either NFAT1-GFP (solid trace) or NFAT4-GFP (dotted trace). (B) Images compare nuclear accumulation of NFAT1- or NFAT4-GFP following stimulation with LTC_4 in cells expressing PV-NLS. (C) Mean data ±SEM from several cells expressing PV-NLS are compared. Each point is the mean of between 14 and 21 cells. (D) Cytoplasmic and nuclear Ca^{2+} are compared for a cell stimulated with 50 nM LTC_4. (E) The number of oscillations per 200 seconds bin are presented. (F) The amplitude of each oscillation in the nuclear and cytoplasmic compartments are plotted against oscillation number. Error bars are contained within the symbols. Each graph is the average of 11-14 cells from 2 independent experiments. (G-I) As in panels D-F, but 2 nM LTC_4 was the stimulus. Each graph depicts mean data ±SEM from 13-21 cells from 3 independent experiments. In panels D-I, PV-NLS was not expressed.

Figure S6. PV-NLS suppresses NFAT4 nuclear accumulation but not that of NFAT1 in cells co-expressing both transcription factors, related to Figure 5. (A) A control cell expressing NFAT1-GFP and NFAT4-cherry was stimulated with thapsigargin and nuclear accumulation is shown after 40 minutes stimulation. (B) Only NFAT1-GFP accumulates in the nucleus after stimulation with thapsigargin in a cell expressing PV-NLS. After 40 minutes stimulation with thapsigargin, the cell was then challenged with 2 μM ionomycin, which led to NFAT4-cherry movement into the nucleus. (C) Mean data from several independent experiments are compared. N1 and N4 denote NFAT1 and NFAT4, respectively. The open bar above N4/PV-NLS shows the subsequent movement after stimulation with ionomycin for 20 minutes. Error bars denote SEM.

Figure S7. Simulation of NFAT1 and NFAT4 nuclear dynamics following CRAC channel activation, related to Figures 1, 5

and 6. (A) The simplified cartoon depicts NFAT movement into and out of the nucleus and shows the differential equations that were used to simulate NFAT nuclear dynamics. The individual rates for NFAT1 were quantified in Kar et al., 2011. Rates for NFAT4 were obtained from Figure 1. The only difference between NFAT1 and NFAT4 was the nuclear export rate, k_4. (B) The graph compares simulated movement of NFAT1 and NFAT4 following low frequency Ca^{2+} pulse of 30 seconds (applied every 15 minutes). A 30 seconds pulse was used to approximate the duration of a Ca^{2+} oscillation in response to cysLT1 receptor activation. (C) In this simulation, Ca^{2+} pulses were applied every 3 minutes. (D) The simulation shows how a change in only the nuclear export rate, k_4, profoundly affects NFAT nuclear accumulation. Control k_4 corresponds to NFAT1. Increasing export rate, by increasing k4, leads to an exponential increase in NFAT export. 7k4 represents NFAT4, as measured in our experiments. In the simulation, Ca^{2+} pulses of 30 seconds duration were applied, but at different intervals (ranging from once every 30 seconds to once every 20 minutes). The simulations were done with Dr Gary Mirams from the Department of Computer Science at Oxford.
