# Supplementary material for: Distinct Spatial Ca2+ Signatures Selectively Activate Different NFAT Transcription Factor Isoforms
Source: Mol Cell. 2015 Apr 16;58(2):232–43. doi: 10.1016/j.molcel.2015.02.027 (PMC4405353; doi:10.1016/j.molcel.2015.02.027)
Supplement: Document S2. Article plus Supplemental Information [file mmc2.pdf]

Molecular Cell

Article

# Distinct Spatial $Ca^{2+}$ Signatures Selectively Activate Different NFAT Transcription Factor Isoforms

## Graphical Abstract

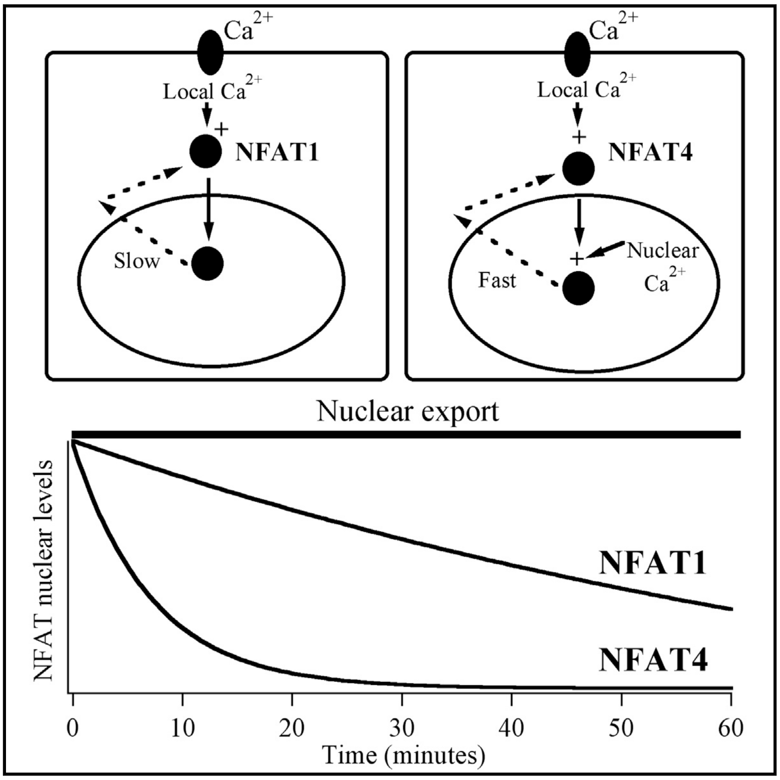


## Authors

Pulak Kar, Anant B. Parekh

## Correspondence

anant.parekh@dpag.ox.ac.uk

## In Brief

Protein isoforms are often co-expressed in cells, occupy the same spatial domain, and are activated by the same intracellular signal. How selective recruitment of one isoform occurs is unclear. Here, we have compared the activation of NFAT1 and NFAT4, two closely related vertebrate transcription factors. We report that the different isoforms are stimulated by distinct sub-cellular $Ca^{2+}$ signatures. NFAT1 has a private line of communication with plasma membrane $Ca^{2+}$ channels, whereas NFAT4 additionally requires a nuclear $Ca^{2+}$ rise.

## Highlights

- Cytoplasmic $Ca^{2+}$ activates NFAT1 and NFAT4 transcription factors
- NFAT1 is stimulated by $Ca^{2+}$ microdomains near plasma membrane $Ca^{2+}$ channels
- NFAT4 requires both $Ca^{2+}$ microdomains and a nuclear $Ca^{2+}$ rise
- Different $Ca^{2+}$ dependencies impart distinct NFAT nuclear residency times

Kar & Parekh, 2015, Molecular Cell *58*, 232–243
April 16, 2015 ©2015 The Authors
http://dx.doi.org/10.1016/j.molcel.2015.02.027

CrossMark
CellPress

Molecular Cell
Article

# Distinct Spatial $Ca^{2+}$ Signatures Selectively Activate Different NFAT Transcription Factor Isoforms

Pulak Kar[1] and Anant B. Parekh[1,*]
[1]Department of Physiology, Anatomy and Genetics, University of Oxford, Parks Road, Oxford OX1 3PT, UK
*Correspondence: anant.parekh@dpag.ox.ac.uk
http://dx.doi.org/10.1016/j.molcel.2015.02.027
This is an open access article under the CC BY license (http://creativecommons.org/licenses/by/4.0/).

## SUMMARY

**Protein isoforms are widely expressed in biological systems. How isoforms that co-exist within the same sub-cellular domain are differentially activated remains unclear. Here, we compare the regulatory mechanism of two closely related transcription factor isoforms, NFAT1 and NFAT4, that migrate from the cytoplasm to the nucleus following the increase in intracellular $Ca^{2+}$ that accompanies the opening of store-operated Orai1/CRAC channels. We demonstrate that NFAT1 has a private line of communication with Orai1, activating in response to $Ca^{2+}$ microdomains near the open channels. By contrast, NFAT4 stimulation requires both local $Ca^{2+}$ entry and a nuclear $Ca^{2+}$ rise. We mapped differences in nuclear location to amino acids within the SP-3 motif of the NFAT regulatory domain. The different $Ca^{2+}$ dependencies enable agonists to recruit different isoform combinations as stimulus strength increases. Our study uncovers a mechanism whereby co-existing cytoplasmic transcription factor isoforms are differentially activated by distinct sub-cellular $Ca^{2+}$ signals.**

## INTRODUCTION

Many signaling proteins have multiple isoforms that are often co-expressed in the same cell. Isoforms can arise from different genes or through alternative splicing of the same gene. The fact that green algae, yeast, *Caenorhabditis elegans*, and vertebrates all express protein isoforms reinforces the view that this tight evolutionary conservation underlies an important, isoform-specific biological function. Critical questions are therefore how some isoforms can be activated, but not others, when they are co-expressed and how different isoforms evoke distinct effects. A partial answer has been provided by the finding that isoforms of protein kinases are corralled to different sub-cellular locations (Mochly-Rosen and Gordon, 1998), controlling only those substrates constrained within the immediate vicinity.

A more fundamental problem arises when protein isoforms share the same spatial domain and are activated by the same intracellular messenger. How can one isoform now be activated selectively and how might it gain access to targets that other isoforms are excluded from? This issue is nicely encapsulated by the NFAT family of transcription factors, which are essential for vertebrate development, differentiation, and function. Four members of the NFAT family (NFAT1–4) are activated by cytoplasmic $Ca^{2+}$ and are encoded by highly homologous genes (Hogan et al., 2003). In resting cells, NFAT proteins are extensively phosphorylated and reside in the cytoplasm. Upon stimulation with agonists that increase intracellular $Ca^{2+}$, these transcription factors are dephosphorylated by $Ca^{2+}$-calmodulin activated protein phosphatase calcineurin, which exposes a nuclear localization sequence and thus enables the protein to translocate to the nucleus where it regulates gene expression (Müller and Rao, 2010; Wu et al., 2007).

In immune cells, NFAT activation is tightly linked to $Ca^{2+}$ entry through store-operated $Ca^{2+}$ release-activated $Ca^{2+}$ (CRAC) channels (Feske et al., 2006; Hogan et al., 2010), and NFAT reporter gene expression is dependent on $Ca^{2+}$ influx (Kar et al., 2012b; Negulescu et al., 1994). CRAC channels open following a fall in $Ca^{2+}$ from within the ER and control a plethora of cellular functions including secretion, gene expression, and regulation of growth and proliferation (Parekh and Putney, 2005). The channels are gated by stromal interaction molecule 1 (STIM1) and STIM2, which are ER $Ca^{2+}$ sensors (Liou et al., 2005; Zhang et al., 2006). As the ER loses $Ca^{2+}$, STIM proteins form multimeric complexes, which then migrate across bulk ER to occupy specialized regions of ER located below the plasma membrane (Hogan et al., 2010; Lewis, 2007; Parekh, 2010). At these sites, STIM proteins bind to and directly activate plasmalemmal Orai1 proteins, which comprise the pore-forming subunit of the CRAC channel (Prakriya et al., 2006; Vig et al., 2006; Yeromin et al., 2006). In RBL-1 cells, a model system for mast cell research, and HEK293 cells, NFAT1 translocation to the nucleus in response to physiological levels of stimulation of endogenous cysteinyl leukotriene type I (cysLT1) receptors is driven by spatially restricted $Ca^{2+}$ signals, called $Ca^{2+}$ microdomains, near open CRAC channels rather than by a global $Ca^{2+}$ rise (Kar et al., 2011, 2012b). The microdomains extend just a few nanometers from the CRAC channel mouth but have privileged access to the NFAT pathway because of the formation of a store-dependent membrane complex that brings calcineurin and a pool of cellular NFAT very close to the $Ca^{2+}$ channel (Kar et al., 2014).

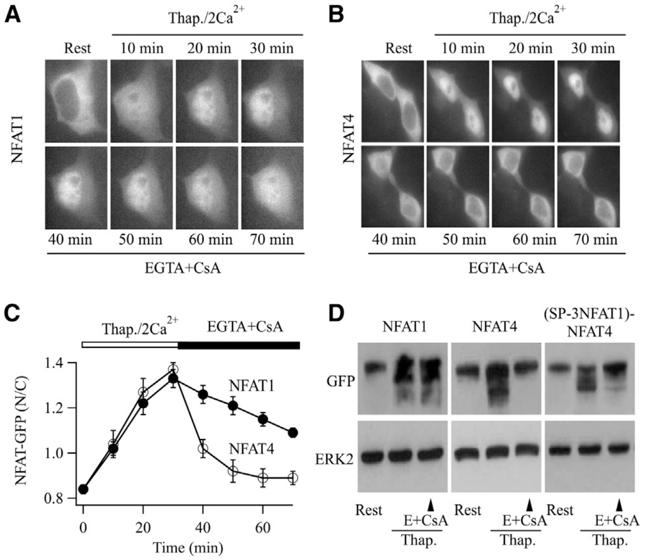


**Figure 1. Different NFAT Isoforms Exhibit Distinct Nuclear Export Kinetics**

(A) Images show the time course of NFAT1-GFP migration into the nucleus following stimulation with 2 μM thapsigargin in external solution containing 2 mM $Ca^{2+}$. After 30 min stimulation, cells were exposed to $Ca^{2+}$-free solution containing 0.1 mM EGTA and 1 μM cyclosporine A (depicted as EGTA+CsA), conditions that result in unidirectional NFAT export from the nucleus (Kar et al., 2011).

(B) Nuclear dynamics of NFAT4-GFP are shown, under identical conditions to those described in (A).

(C) Aggregate data are compared. Each point is the average of between 17 and 22 individual cells and is represented as mean ± SEM.

(D) Gel shifts compare the extent of NFAT1, NFAT4, and (SP-3NFAT1)NFAT4-GFP rephosphorylation following stimulation with thapsigargin for 30 min to induce dephosphorylation. Rephosphorylation was then promoted by exposing the cells to $Ca^{2+}$-free solution and cyclosporine A for 20 min, after which cell lysates were obtained. Similar results were obtained in two further independent experiments.

NFAT1 and NFAT4 are often co-expressed in cells, occupy the same cytoplasmic location, and are activated by intracellular $Ca^{2+}$. Nevertheless, they have recently been found to exhibit marked differences in nuclear translocation dynamics. NFAT4 migration into, and export from, the nucleus was reported to be ~5–10 times faster than the corresponding rates for NFAT1 (Yissachar et al., 2013). Because NFAT1 and NFAT4 are strongly expressed in immune cells, these differences in isoform dynamics might increase the temporal bandwidth for information processing in the immune system. However, the mechanistic basis for the different nuclear kinetics between the two transcription factor isoforms is unknown.

The sub-cellular profile of the $Ca^{2+}$ signal is critical in selective activation of downstream targets. Here, we compared the dependence of NFAT1 and NFAT4 nuclear accumulation on both local and global cytoplasmic $Ca^{2+}$. We find that NFAT1 is tightly coupled to Orai1, whereas NFAT4 relies on a form of coincidence detection, requiring both local $Ca^{2+}$ entry and a nuclear $Ca^{2+}$ rise. Our study uncovers a mechanism whereby co-existing transcription factor isoforms are activated by distinct sub-cellular $Ca^{2+}$ signals.

## RESULTS

### Hysteresis in Nuclear NFAT Dynamics

Activation of CRAC channels following stimulation of either G protein coupled cysLT1 receptors or tyrosine kinase-linked FCεRI receptors or by exposure to the sarco-ER $Ca^{2+}$ATPase pump blocker thapsigargin all led to prominent movement of NFAT1-GFP from the cytoplasm into the nucleus in RBL-1 cells (Kar et al., 2012a, 2011). We compared the nuclear dynamics of NFAT1 with NFAT4 in response to the same stimulus, a maximally effective dose of thapsigargin, in cells expressing either NFAT1-GFP or NFAT4-GFP. In the presence of thapsigargin, NFAT1-GFP (Figure 1A) and NFAT4-GFP (Figure 1B) both migrated into the nucleus with similar kinetics (half-time for nuclear import was 11.1 ± 2.4 min for NFAT1-GFP and 12.2 ± 2.3 min for NFAT4-GFP, respectively; $p > 0.4$; Figure 1C). Following steady-state nuclear accumulation, we measured the unidirectional nuclear export of NFAT by removing external $Ca^{2+}$ and simultaneously blocking calcineurin with cyclosporine A (Kar et al., 2011). Under these conditions, NFAT1-GFP efflux was slow (Figures 1A and 1C; time-constant of 73.5 ± 8.8 min). By contrast, NFAT4-GFP export was ~10 times faster (Figures 1B and 1C; time constant of 7.8 ± 2.4 min; $p < 0.001$).

Similar results were obtained with a physiologically relevant stimulus. Activation of cysLT1 receptors with leukotriene $C_4$ ($LTC_4$) increases $InsP_3$ levels and generates cytoplasmic $Ca^{2+}$ oscillations that are accompanied by CRAC channel opening (Di Capite et al., 2009). Application of $LTC_4$ led to migration of NFAT1 (Figure S1A) and NFAT4 (Figure S1C) into the nucleus, with similar kinetics (Figures S1B and S1D). In the continued presence of agonist, only a small fraction of either transcription factor exited the nucleus (Figure S1B). Following nuclear accumulation, we removed $LTC_4$ and applied the cysLT1 receptor antagonist montelukast. Whereas most of the NFAT1 remained within the nucleus 30 min later (Figure S1B), NFAT4 was exported quickly (Figure S1D). The kinetics of the export of either NFAT1 or NFAT4 were not affected by the additional presence of cyclosporine A (Figures S1A and S1C; aggregate data summarized in Figures S1B and S1D).

Nuclear NFAT is rephosphorylated by protein kinases, which leads to its export to the cytoplasm. We compared the extent of rephosphorylation of NFAT isoforms by tracking the gel shift that occurs when dephosphorylated NFAT is rephosphorylated. In resting cells, both NFAT1-GFP and NFAT4-GFP were extensively phosphorylated and $Ca^{2+}$ influx through CRAC channels dephosphorylated each isoform, resulting in prominent gel shifts (Figure 1D, top). After 30 min of sustained $Ca^{2+}$ entry, a condition that results in maximal accumulation of NFAT within the nucleus (Figure 1C), we removed external $Ca^{2+}$ and added cyclosporine A to prevent further dephosphorylation. We then measured the extent of rephosphorylation 20 min later. Consistent with the data obtained using GFP-tagged NFAT constructs, we found significantly more rephosphorylation of NFAT4 compared with NFAT1 at this time point (Figure 1D).

The kinetic analyses in Figures 1 and S1 reveal interesting differences between NFAT1 and NFAT4, in response to the same level of stimulation with either thapsigargin or $LTC_4$. NFAT1 shows marked hysteresis, with nuclear export ~6 times slower

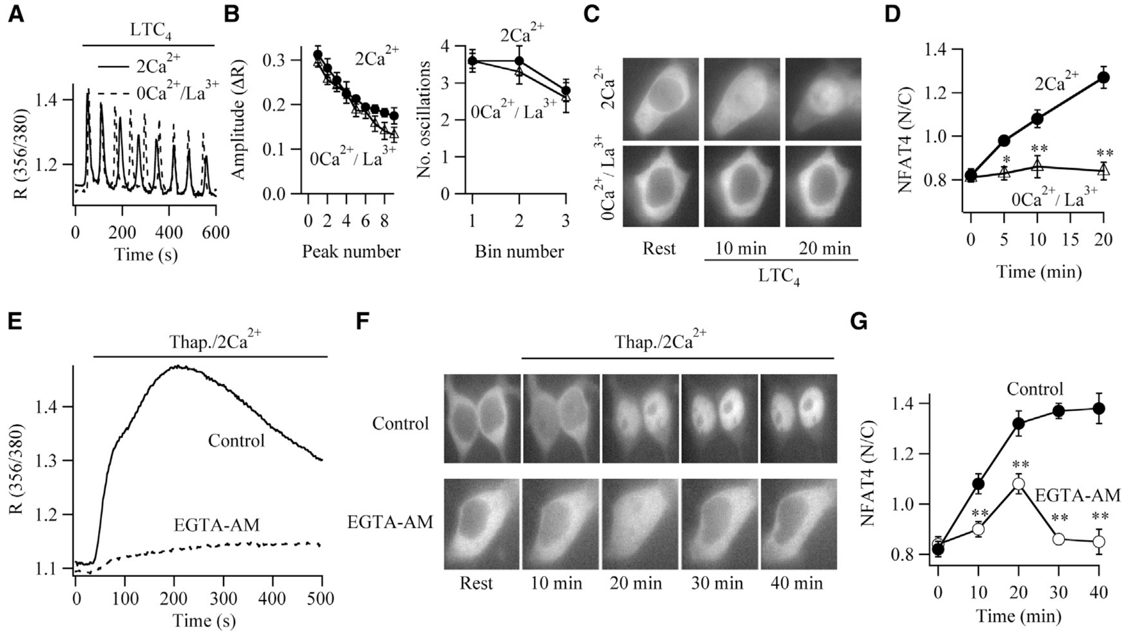


**Figure 2. $Ca^{2+}$ Microdomains near Open CRAC Channels Activate NFAT4**

(A) Cytoplasmic $Ca^{2+}$ oscillations in response to stimulation of leukotriene receptors with 160 nM $LTC_4$ in either 2 mM external $Ca^{2+}$ or $Ca^{2+}$-free external solution containing 1 mM $La^{3+}$ are compared.

(B) Graphs summarize data (mean ± SEM) from several experiments. The left graph shows the amplitude of each oscillation plotted against the oscillation (peak) number. The right graph depicts the number of oscillations recorded every 200 s bin after stimulation.

(C) Images compare NFAT4-GFP migration to the nucleus in cells stimulated with $LTC_4$ either in the presence of external $Ca^{2+}$ or in $0Ca^{2+}/La^{3+}$-containing external solution.

(D) Mean data ± SEM are compared. Each point is the average of between 9 and 14 cells.

(E) Loading the cytoplasm with EGTA (by incubating cells with EGTA-AM) significantly reduced the cytoplasmic $Ca^{2+}$ rise evoked by thapsigargin.

(F) Images compare NFAT4-GFP nuclear accumulation at different times after stimulation with thapsigargin between control cells and a cell exposed to EGTA-AM.

(G) The time course of nuclear NFAT4-GFP is compared between EGTA-loaded cells (open circles) and controls (filled circles) from the same preparations and used on the same days. Each point reflects between 12 and 16 cells. ** denotes $p < 0.01$. Data are represented as mean ± SEM.

than import. By contrast, NFAT4 has similar import and export rates. NFAT4 nuclear residency is therefore relatively better coupled to the duration of the cytoplasmic $Ca^{2+}$ signal. NFAT1 resides within the nucleus long after $Ca^{2+}$ entry has terminated, thereby imparting a form of short-term memory to NFAT-dependent gene expression (Kar et al., 2012b).

## NFAT4 Residency in the Nucleus Requires Local and Global $Ca^{2+}$

We designed experiments to address the mechanistic basis for the different nuclear export rates between NFAT1-GFP and NFAT4-GFP. NFAT1 translocation to the nucleus is triggered by $Ca^{2+}$ microdomains near open CRAC channels. Two lines of evidence suggest that local $Ca^{2+}$ entry also regulates NFAT4 migration. First, stimulation of HEK293 cells expressing cysLT1 receptors and NFAT4-GFP with $LTC_4$ evoked a series of cytoplasmic $Ca^{2+}$ oscillations that reflected regenerative $Ca^{2+}$ release from the stores accompanied by $Ca^{2+}$ influx through CRAC channels (Figure 2A). Robust translocation of NFAT4-GFP into the nucleus was observed (Figures 2C, top, and 2D). Repetitive $Ca^{2+}$ oscillations to $LTC_4$ can be evoked in the absence of $Ca^{2+}$ influx by the blocking of the plasma membrane $Ca^{2+}$ATPase pumps with $La^{3+}$ (Bird and Putney, 2005; Di Capite et al., 2009). Under these conditions, no $Ca^{2+}$ transport occurs across the plasma membrane and so $Ca^{2+}$ release is sequestered back into the stores in readiness for the next oscillatory cycle. Although $La^{3+}$-treated cells evoked $Ca^{2+}$ oscillations of similar amplitude and frequency to those seen in the presence of external $Ca^{2+}$ (Figures 2A and 2B), NFAT4-GFP translocation was undetectable (Figures 2C and 2D). Second, we loaded the cytoplasm with the $Ca^{2+}$ chelator EGTA, which prevents global $Ca^{2+}$ from rising but is too slow to interfere with $Ca^{2+}$ microdomains near open CRAC channels (Neher, 1998; Parekh, 2008). The large cytoplasmic $Ca^{2+}$ rise evoked by thapsigargin was suppressed in EGTA-loaded cells (Figure 2E), but NFAT4-GFP still migrated to the nucleus (Figures 2F and 2G).

We noticed that nuclear accumulation of NFAT4-GFP in the presence of cytoplasmic EGTA was less and considerably more transient than that seen in its absence (Figure 2G). This was not the case for NFAT1, where nuclear dynamics was unaffected by cytoplasmic EGTA (Figure S2) (Kar et al., 2011). Although local $Ca^{2+}$ influx triggers NFAT1 and NFAT4 movement, these results reveal that a global $Ca^{2+}$ rise is needed to maintain NFAT4-GFP within the nuclear compartment.

CellPress

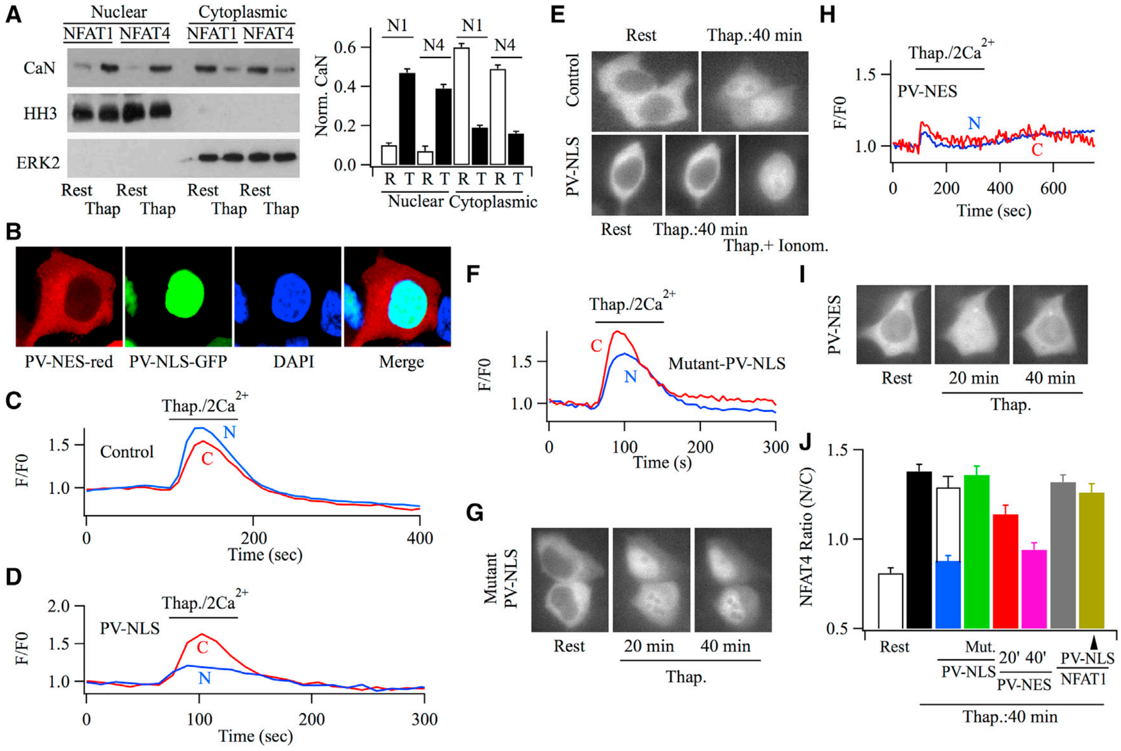


**Figure 3. Nuclear $Ca^{2+}$ Maintains NFAT4 within the Nucleus**

(A) Calcineurin translocates from the cytoplasm to the nucleus after CRAC channel activation. Histone H3 (HH3) was used as a marker for the nuclear fraction and ERK2 was one for the cytoplasmic fraction. Cells were stimulated with thapsigargin for 20 min before lysis. In between the nuclear and cytoplasmic lanes is the marker lane. The histogram summarizes results from three independent experiments. N1, NFAT1; N4, NFAT4; R, resting state; T, thapsigargin stimulation.

(B) Confocal images show the spatial distribution of the parvalbumin constructs used.

(C) Cytoplasmic and nuclear $Ca^{2+}$ measurements are compared. A pulse of $Ca^{2+}$ was applied after store depletion with thapsigargin.

(D) Cytoplasmic and nuclear $Ca^{2+}$ are shown after expression of PV-NLS (untagged).

(E) Images compare NFAT4-GFP accumulation in the nucleus between two control (mock-transfected) cells and one expressing untagged PV-NLS. The thap.+ ionom. image was taken 20 min after ionomycin was applied.

(F) Cytoplasmic and nuclear $Ca^{2+}$ are compared in a cell expressing mutant PV-NLS, which localizes to the nucleus but cannot bind $Ca^{2+}$.

(G) Images show movement of NFAT4-GFP into the nucleus after stimulation of cells expressing mutant PV-NLS.

(H) Cytoplasmic and nuclear $Ca^{2+}$ measurements are shown for a cell expressing PV-NES.

(I) NFAT4-GFP nuclear accumulation is shown for a cell expressing PV-NES.

(J) Aggregate data from several experiments are compared. The white bar on top of the bar for PV-NLS shows the extent of NFAT4-GFP accumulation in response to ionomycin (2 $\mu$M) in cells expressing PV-NLS. In these experiments, cells were first stimulated with thapsigargin and NFAT4-GFP accumulation measured (blue bar). Ionomycin was then applied (as in E) and translocation quantified 20 min later. The two bars for PV-NES summarize NFAT4-GFP nuclear levels after 20 and 40 min stimulation with thapsigargin. Data in the histogram reflect mean ± SEM.

## Nuclear $Ca^{2+}$ Maintains NFAT4 in the Nucleus

The different export rates of NFAT1 and NFAT4 are likely to reflect disparities in isoform regulation within the nucleus. Three types of protein kinase are known to phosphorylate nuclear NFAT, resulting in exposure of a nuclear export sequence. These kinases are dual-specificity tyrosine-phosphorylation regulated kinases (DYRKs), casein kinase 1, and glycogen synthase kinase 3. DYRKs directly phosphorylate the serine-proline repeat (SP-3) motif, which primes for further phosphorylation of the SP-2 and serine-rich region 1 (SRR-1) by the other kinases (Gwack et al., 2006). No $Ca^{2+}$-dependent regulation of these kinases has been reported. We therefore considered that global $Ca^{2+}$ maintained NFAT4-GFP in the nucleus not through blocking of phosphorylation but by sustaining dephosphorylation. Although a cytoplasmic protein, calcineurin can translocate into the nucleus, where it counters nuclear NFAT phosphorylation (Al-Daraji et al., 2002; Shibasaki et al., 1996). To mimic the experimental conditions we used above, we transfected cells with either NFAT1- or NFAT4-GFP and then measured the distribution of endogenous calcineurin between cytoplasmic and nuclear compartments. In cells expressing either NFAT1- or NFAT4-GFP, western blot analysis revealed that the majority of calcineurin was in the cytoplasmic fraction at rest but significant movement into the nucleus occurred after stimulation with thapsigargin (Figure 3A). The time course of calcineurin migration was ~3-fold slower than that of either NFAT-GFP isoform, implying that the two proteins did not move as a complex. Because a global cytoplasmic $Ca^{2+}$ rise can rapidly propagate into the nucleus (Oliveira et al., 2014), we hypothesized that it was nuclear $Ca^{2+}$ that maintained NFAT4 in the nucleus. To test this, we transfected cells

with the $Ca^{2+}$-binding protein parvalbumin (PV) that had been engineered to express only in the nucleus by virtue of insertion of a nuclear localization sequence (NLS) (referred to as PV-NLS). PV-NLS-GFP was retained exclusively in the nucleus (Figure 3B), co-localizing with the nuclear stain DAPI. To confirm a $Ca^{2+}$ buffering action within the nucleus, we measured nuclear $Ca^{2+}$ with fluo-4 using confocal microscopy. Stimulation with a $Ca^{2+}$ pulse after thapsigargin treatment evoked a rise in both cytoplasmic and nuclear $Ca^{2+}$ (Figure 3C; Figure S3A; mean data are shown in Figure S3B). In cells transfected with PV-NLS (an untagged construct that was used to prevent interference with the fluo-4 signal), the cytoplasmic $Ca^{2+}$ rise in response to thapsigargin was unaffected, but the nuclear $Ca^{2+}$ increase was substantially reduced (Figure 3D; Figure S3B). PV-NLS therefore effectively buffers nuclear $Ca^{2+}$. To examine whether nuclear $Ca^{2+}$ was required to maintain NFAT4 within the nucleus, we co-expressed PV-NLS (untagged) with NFAT4-GFP and stimulated cells with thapsigargin. NFAT4-GFP accumulation within the nucleus was now virtually undetectable (Figure 3E; corresponding controls from the same preparations of cells, but not transfected with PV-NLS, are shown in Figure 3E, top). Aggregate data from several independent experiments are summarized in Figure 3J. Two pieces of evidence demonstrated that the inhibitory action of PV-NLS was due to its ability to buffer nuclear $Ca^{2+}$. First, we raised cytoplasmic $Ca^{2+}$ to high levels by stimulation with the $Ca^{2+}$ ionophore ionomycin, a condition that would be expected to saturate the $Ca^{2+}$-binding ability of PV-NLS, enabling nuclear $Ca^{2+}$ to rise. Whereas low doses of ionomycin (nM– hundreds of nM range) selectively deplete stores and thus open CRAC channels, higher concentrations ($\mu$M) raise cytoplasmic $Ca^{2+}$ mainly through direct $Ca^{2+}$ entry by the ionophore. In intact RBL-1 cells, for example, the sustained $Ca^{2+}$ response to 2 $\mu$M ionomycin is weakly affected by $La^{3+}$, a CRAC channel blocker (Di Capite et al., 2009). Consistent with this, $Ca^{2+}$ entry in response to 2 $\mu$M ionomycin was unaffected by the CRAC channel blocker BTP2 (the peak $Ca^{2+}$ rise [$\Delta$R] after readmission of 2 mM $Ca^{2+}$ to cells challenged with ionomycin in $Ca^{2+}$-free solution was 1.36 ± 0.12 [control], and this became 1.29 ± 0.13 after 15 min pre-exposure to BTP2, $p > 0.2$). However, BTP2 inhibited store-operated $Ca^{2+}$ entry by 82% ± 9% when applied after $Ca^{2+}$ influx had been evoked by thapsigargin. Although stimulation with thapsigargin in cells expressing PV-NLS caused little NFAT4 translocation, subsequent exposure to 2 $\mu$M ionomycin in these same cells rescued NFAT4-GFP accumulation within the nucleus (Figure 3E; open bar above PV-NLS in Figure 3J). Application of ionomycin also rescued cytoplasmic and nuclear $Ca^{2+}$ signals in cells expressing PV-NLS (Figures S3A and S3B). Second, we expressed a mutant PV-NLS construct in which a critical glutamic acid residue at position 12 of each $Ca^{2+}$-binding loop had been mutated to a valine (E62V, E101V) so that mutant PV-NLS was unable to bind $Ca^{2+}$ (Pusl et al., 2002). Expression of this mutant had no effect either on the nuclear $Ca^{2+}$ rise (Figure 3F; Figure S3C) or NFAT4-GFP accumulation within the nucleus (Figures 3G and 3J). In contrast to NFAT4, expression of PV-NLS had no inhibitory effect on nuclear accumulation of NFAT1-GFP (Figures 3J and S2).

Following elevation of $InsP_3$, nuclear $Ca^{2+}$ can increase independently of global cytoplasmic $Ca^{2+}$, an effect arising from the presence of type II $InsP_3$ receptors in the nuclear membrane (Leite et al., 2003). In our experiments, $Ca^{2+}$ entry through CRAC channels was used to stimulate NFAT and nuclear $Ca^{2+}$ would therefore follow the rise in global cytoplasmic $Ca^{2+}$. Consistent with this, expression of an mcherry-tagged PV construct that contained a nuclear export sequence (NES) (referred to as cytosolic PV-NES) resulted in a predominantly cytoplasmic distribution (Figure 3B) and the rise in both cytoplasmic and nuclear $Ca^{2+}$ following stimulation was inhibited (Figure 3H; Figure S3C). NFAT4-GFP accumulation within the nucleus was also reduced by PV-NES, and this became more prominent at later times (Figures 3I and 3J).

## NFAT1 and NFAT4 Activation Exhibit Different Sensitivity to Agonist

The finding that NFAT4, but not NFAT1, requires a rise in nuclear $Ca^{2+}$ suggests that the two isoforms might have different dependencies on agonist intensity. Because NFAT1 is linked tightly to $Ca^{2+}$ microdomains near CRAC channels, modest stimulation of cell-surface receptors, which opens a fraction of the channels, should result in significant nuclear migration. By contrast, such modest stimulation is unlikely to raise global and therefore nuclear $Ca^{2+}$ sufficiently for NFAT4 to accumulate within the nucleus. To test whether the isoforms exhibited different stimulus intensity dependencies, we expressed cysLT1 receptors together with either NFAT1- or NFAT4-GFP in HEK293 cells and then activated the receptors over a range of $LTC_4$ concentrations. Receptor activation with 160 nM $LTC_4$ evoked repetitive $Ca^{2+}$ oscillations, which led to prominent movement of both NFAT1 and NFAT4 into the nucleus (data not shown). Stimulation with either 50 nM (Figures 4A–4D) or 20 nM (Figures 4E–4H) $LTC_4$ also generated several $Ca^{2+}$ oscillations and subsequent accumulation of either NFAT-GFP isoform in the nucleus. Stimulation with 5 nM (Figures 4I–4L) or 2 nM (Figures 4M–4O) $LTC_4$ evoked a series of $Ca^{2+}$ oscillations, albeit of smaller amplitude and fewer in number when compared with the larger doses, but these still triggered the movement of NFAT1-GFP into the nucleus. By contrast, NFAT4-GFP failed to migrate to the nucleus at these low levels of receptor stimulation. A comparison of the dependences of NFAT-GFP isoform migration on stimulus intensity revealed that NFAT1 activation was >5-fold more sensitive to cysLT1 receptor activation than NFAT4 (Figure 4P).

One potential concern with these experiments is that cysLT1 receptor expression or its downstream signaling might differ between cells transfected with either NFAT1- or NFAT4-GFP. The frequency of $Ca^{2+}$ oscillations is very sensitive to receptor levels, phospholipase C activity, SERCA pump expression, and mitochondrial $Ca^{2+}$ uptake. We therefore compared the amplitude and frequency of $Ca^{2+}$ oscillations between cells expressing cysLT1 receptors alone, cysLT1 receptors with NFAT1, and cysLT1 receptors with NFAT4. These oscillatory components were virtually identical over a range of $LTC_4$ concentrations (Figure S4). Moreover, the amplitudes and frequencies of $Ca^{2+}$ oscillations elicited by 2 nM were similar between cells expressing either cysLT1 receptors alone, receptors and NFAT1, or receptors and NFAT4 (Figures S4A and S4D). Responses to 50 nM $LTC_4$ were also similar (Figures S4A and S4D), demonstrating

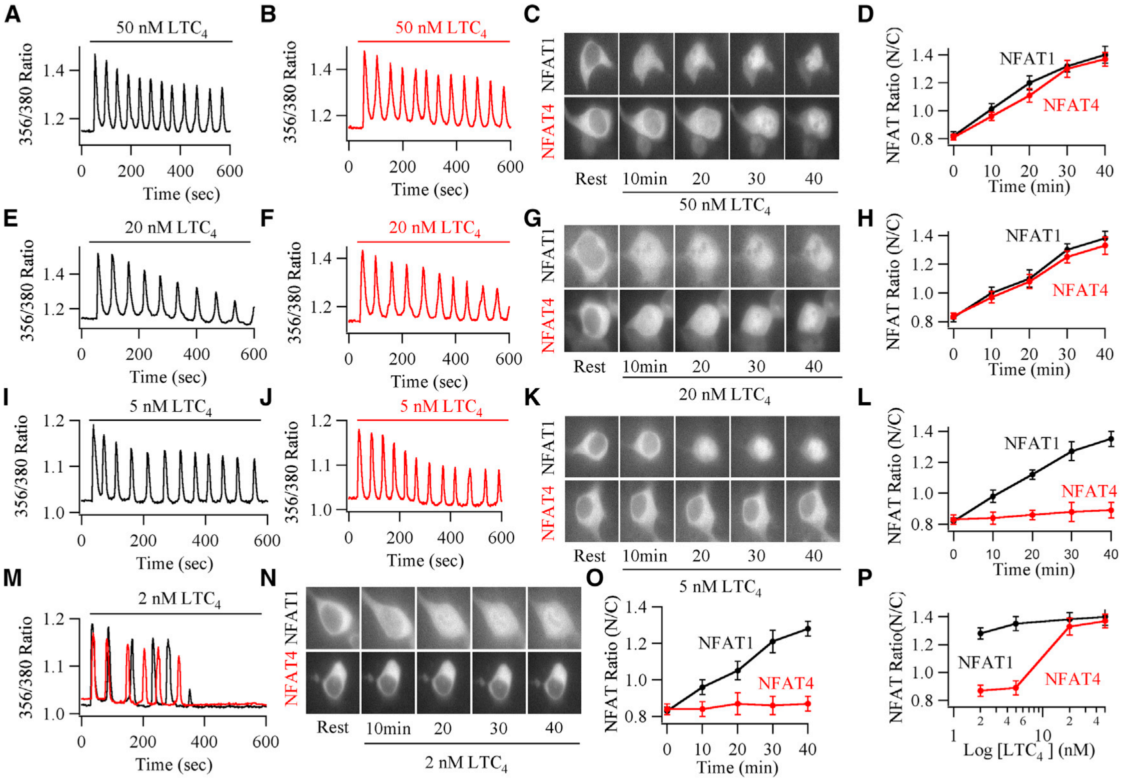


**Figure 4. NFAT Isoforms Exhibit Different Activation Thresholds**

(A) Numerous $Ca^{2+}$ oscillations are evoked by 50 nM $LTC_4$ in cells expressing NFAT1-GFP.
(B) A similar pattern of $Ca^{2+}$ oscillations is induced by $LTC_4$ in cells expressing NFAT4-GFP.
(C) Isoform accumulation within the nucleus is compared following stimulation with $LTC_4$.
(D) Data (mean ± SEM) are compared. Each point represents between 6 and 14 cells.
(E–H) Identical profile to (A)–(D), but now in the presence of 20 nM $LTC_4$.
(I–L) The effects of 5 nM $LTC_4$ on NFAT1 and NFAT4 are compared, as in (A)–(D).
(M) Cytoplasmic $Ca^{2+}$ oscillations to 2 nM $LTC_4$ are compared between cells expressing NFAT1-GFP (black trace) and NFAT4-GFP (red trace).
(N) Images compare NFAT1-GFP and NFAT4-GFP nuclear accumulation following stimulation with 2 nM $LTC_4$.
(O) Aggregate data are compared. Each point is the average of between 14 and 21 cells.
(P) The dependence of NFAT1 and NFAT4 nuclear accumulation on agonist concentration is compared. Aggregate data are mean ± SEM.

that receptor expression and downstream signaling were comparable for the different cells.

## Nuclear $Ca^{2+}$ Is Required for NFAT4 Migration in Response to Oscillatory $Ca^{2+}$ Signals

We tested the physiological relevance of a nuclear $Ca^{2+}$ rise in distinguishing between activation of the NFAT isoforms by stimulating cells with a low concentration of $LTC_4$ but after expression of PV-NLS.

50 nM $LTC_4$ evoked a similar pattern of cytoplasmic $Ca^{2+}$ oscillations in cells expressing either NFAT1-GFP or NFAT4-GFP together with PV-NLS (Figure S5A). However, only NFAT1 migrated to the nucleus; buffering of nuclear $Ca^{2+}$ suppressed NFAT4 migration in response to cysLT1 receptor activation (Figure S5B; aggregate data are shown in Figure S5C).

Simultaneous measurements of cytoplasmic and nuclear $Ca^{2+}$ showed that robust nuclear $Ca^{2+}$ oscillations were generated in phase with cytoplasmic $Ca^{2+}$ oscillations when cells were stimulated with 50 nM $LTC_4$ (Figures S5D–S5F). However, in the presence of 2 nM $LTC_4$, a concentration that activated NFAT1 but not NFAT4 migration (Figure 4O), weak cytoplasmic $Ca^{2+}$ oscillations were generated, and these were associated with only small and transient nuclear $Ca^{2+}$ signals (Figures S5G–S5I).

## Different NFAT Nuclear Dynamics in Cells Co-expressing NFAT1 and NFAT4

Although the previous experiments showed marked differences in nuclear export between NFAT1 and NFAT4, the studies were carried out in cells expressing either transcription factor alone. To study transcription factor dynamics under identical conditions, we co-transfected cells with NFAT1-GFP and NFAT4-cherry and measured the kinetics of nuclear import and export in response to the same stimuli: thapsigargin or $LTC_4$. In the first set of experiments, we fixed dual-expressing cells at different times after stimulation and measured the sub-cellular distribution of NFAT1 and NFAT4, using confocal microscopy after staining the nucleus with DAPI. At rest, both NFAT1-GFP and NFAT4-cherry were confined to the cytosol (Figure 5A). After stimulation

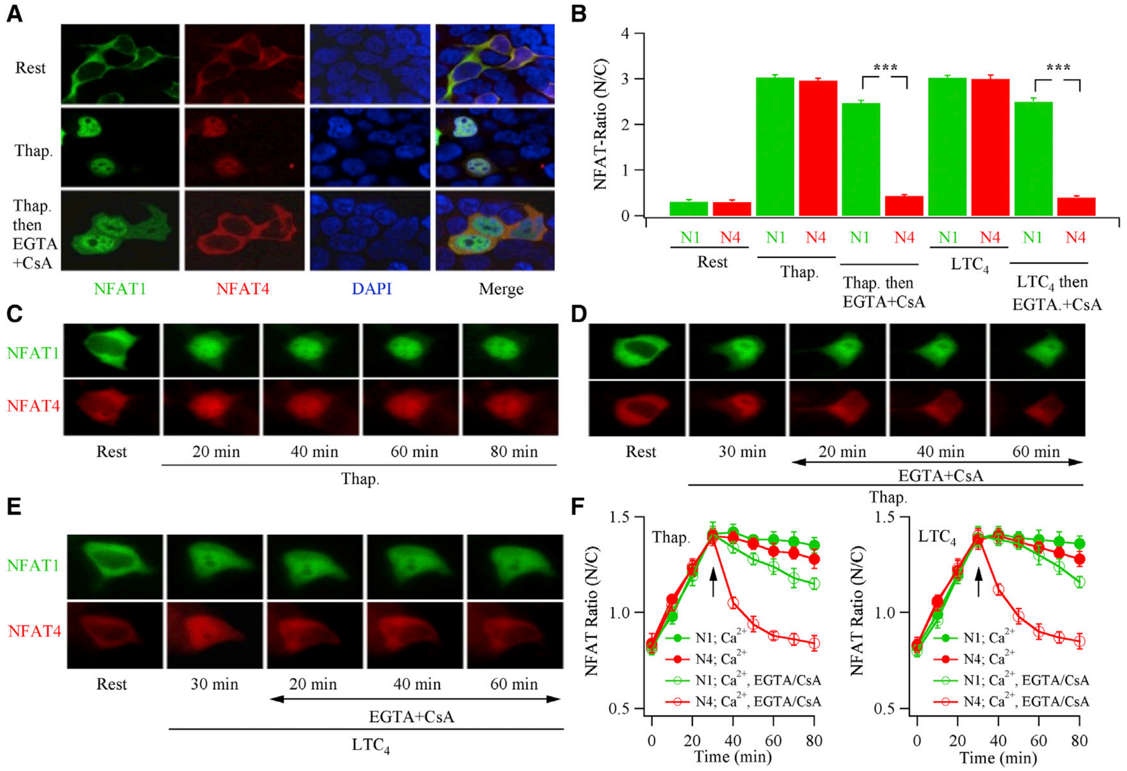


**Figure 5. NFAT Nuclear Dynamics in Cells Co-expressing NFAT1 and NFAT4**

(A) Confocal images compare NFAT1-GFP and NFAT4-cherry distribution in the same cells. Rest denotes the non-stimulated condition, Thap. represents stimulation with thapsigargin for 30 min, and Thap. then EGTA+CsA denotes stimulation with thapsigargin for 30 min followed by exposure to thapsigargin- and $Ca^{2+}$-free solution supplemented with cyclosporine A for 20 min prior to fixing. Nuclei were stained with DAPI.

(B) Aggregate data from experiments as in (A) are described. Each bar represents data (mean ± SEM) from at least 28 cells from 3 coverslips. N1 and N4 denote NFAT1 and NFAT4.

(C) Live cell imaging of NFAT nuclear accumulation is compared in a single cell co-expressing NFAT1-GFP and NFAT4-cherry. Thapsigargin was applied for the times indicated.

(D) NFAT nuclear export is compared in a cell co-expressing NFAT1-GFP (top) and NFAT4-cherry. After 30 min stimulation with thapsigargin, cells were perfused with $Ca^{2+}$-free solution containing 0.1 mM EGTA and cyclosporine A.

(E) A similar experiment to that in (D) is shown, but now the stimulus was 120 nM $LTC_4$.

(F) Aggregate data for cells co-expressing NFAT1-GFP and NFAT4-cherry are compared. Stimulus (thapsigargin or $LTC_4$) was applied immediately after resting images were obtained (time zero). At the arrow, stimulus was removed and cells were exposed to $Ca^{2+}$-free solution containing EGTA and cyclosporine A. For the traces labeled N1;$Ca^{2+}$ and N4;$Ca^{2+}$, the stimulus was maintained (controls). Each point is the average of between 14 and 23 cells, taken from 5 and 9 coverslips. Aggregate data are mean ± SEM.

with thapsigargin for 30 min, both transcription factors had migrated into the nucleus. 20 min after exposure to $Ca^{2+}$-free solution supplemented with cyclosporine A, almost all NFAT4-cherry had returned to the cytoplasm, whereas nuclear NFAT1-GFP levels had barely changed (Figure 5A; mean data are shown in Figure 5B). NFAT4-cherry also migrated out of the nucleus considerably faster than NFAT1 after cells had initially been stimulated with $LTC_4$ instead (Figure 5B).

To study transcription factor kinetics in more detail, we turned to live epifluorescence imaging and measured the movement of NFAT1-GFP and NFAT4-cherry in co-transfected cells over a period of 80 min. Both transcription factors moved into the nucleus with similar kinetics following stimulation with thapsigargin (Figures 5C and 5F, left). However, upon exposure to $Ca^{2+}$-free solution containing cyclosporine A, NFAT4-cherry export was considerably faster than that of NFAT1-GFP (Figures 5D and 5F). Similar results were obtained when $LTC_4$ was used as the trigger instead (Figures 5E and 5F, right). In cells expressing only NFAT4-GFP, the time-constant for export was 7.8 ± 2.4 min (Figure 1), and in dual-expressing cells, NFAT-cherry export was 10.7 ± 1.8 min ($p > 0.1$). Corresponding values for NFAT1-GFP were 73.5 ± 8.8 and 71.2 ± 6.9 min ($p > 0.1$), respectively. This shows that the presence of one tagged NFAT isoform does not significantly affect the dynamics of the other.

We compared the dependence of NFAT1-GFP with NFAT4-cherry on nuclear $Ca^{2+}$ in co-expressing cells. In control cells not transfected with PV-NLS, stimulation with thapsigargin resulted in nuclear accumulation of both NFAT1 and NFAT4 (Figures S6A and S6C). In cells expressing PV-NLS, only NFAT1 moved into the nucleus following stimulation with thapsigargin (Figures S6B and S6C). Subsequent challenge with ionomycin led to NFAT4 translocation to the nucleus (Figures S6B and

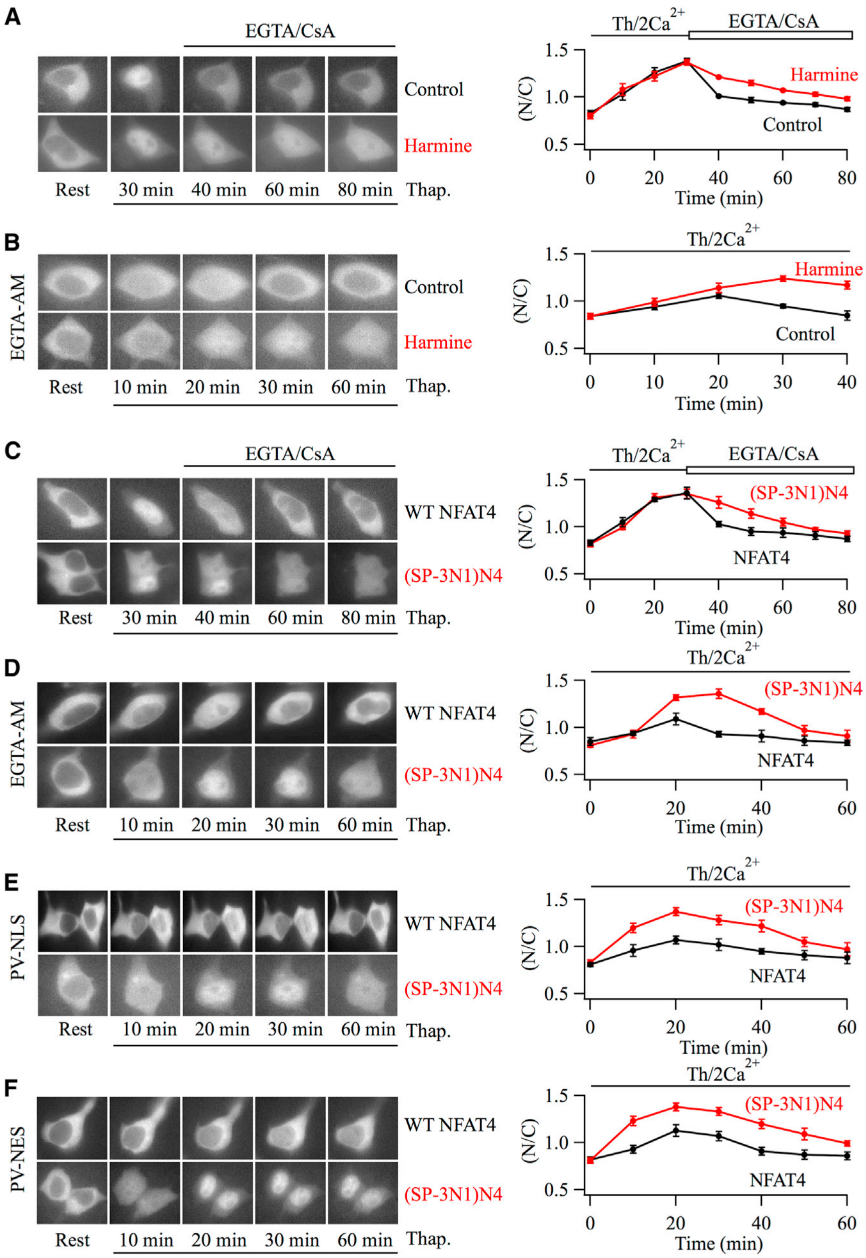


**Figure 6. The SP-3 Motif of NFAT1 Slows Nuclear Export**

(A) Images compare NFAT4-GFP nuclear export kinetics between a control cell and one pre-exposed to harmine (5 μM for 10 min). Aggregate data are summarized on the right. Each point shows the mean ± SEM of between 14 and 21 cells.

(B) NFAT4-GFP nuclear accumulation is compared between a control cell and one pre-exposed to harmine. Both cells were loaded with EGTA-AM prior to stimulation. The graph depicts data (mean ± SEM) from between 11 and 19 cells per point.

(C) Images compare nuclear dynamics of NFAT4-GFP with (SP-3N1)N4, which denotes NFAT4-GFP but now contains the SP-3 domain of NFAT1 instead ((SP-3N1)NFAT4-GFP). Each point in the graph is the mean ± SEM of between 14 and 24 cells.

(D) Nuclear dynamics of NFAT4-GFP and (SP-3N1)NFAT4-GFP are compared in cells loaded with EGTA. Each point on the graph shows mean data (±SEM) from between 7 and 11 cells.

(E) Nuclear movement of NFAT4-GFP is compared with (SP-3N1)N4-GFP in cells expressing PV-NLS. Each data point in the graph depicts mean ± SEM from between 14 and 23 cells.

(F) Nuclear dynamics of NFAT4-GFP and (SP-3N1)N4-GFP are compared in cells expressing PV-NES. Each point reflects mean data ± SEM from between 16 and 25 cells. In (A) and (C), NFAT movement into the nucleus was triggered by stimulating cells with thapsigargin in 2 mM external $Ca^{2+}$ for 30 min before nuclear export was induced by perfusing cells with $Ca^{2+}$-free external solution containing cyclosporine A, for the times shown.

In (B), (D), (E), and (F), cells were stimulated with thapsigargin in the continuous presence of external $Ca^{2+}$.

S6C). These data confirm the different requirements for nuclear $Ca^{2+}$, but now in cells co-expressing both transcription factors.

## The SP-3 Motif in the NFAT Regulatory Domain Contributes to Nuclear Residency

The SP-3 region in the regulatory domain of NFAT proteins is particularly important in regulating nuclear export because phosphorylation by DYRKs within this motif primes for further phosphorylation within the conserved SRR-1 and SP-2 regions (Gwack et al., 2006). We therefore hypothesized that the disparity in nuclear residency between NFAT1 and NFAT4 was due, at least in part, to differences in the respective SP-3 regions. Because DYRKs phosphorylate several serine residues in the SP-3 motif (Okamura et al., 2000), a simple prediction would be that blocking of these kinases should slow nuclear export of NFAT4. Consistent with this, pre-treatment with the DYRK inhibitor harmine (Göckler et al., 2009; Seifert et al., 2008) slowed NFAT4-GFP nuclear export ~3-fold (Figure 6A; mean data are shown on the right; the time-constant for control was 8.5 ± 2.1 min and this increased to 26.7 ± 3.5 min in harmine, $p < 0.01$). In the presence of harmine, however, NFAT4-GFP was still exported ~2.5-fold more rapidly than NFAT1-GFP, suggesting the presence of either harmine-insensitive DYRKs or DYRK-independent phosphorylation sites. A similar slowing of NFAT4 export by harmine was seen after stimulation with $LTC_4$ (Figures S1C and S1D). By contrast, NFAT1 export was only weakly affected by harmine (Figures S1A and S1B). Nuclear NFAT4-GFP accumulation was also increased in EGTA-loaded cells when harmine was present (Figure 6B). To test the involvement of the SP-3 motif more directly, we used a chimera in which

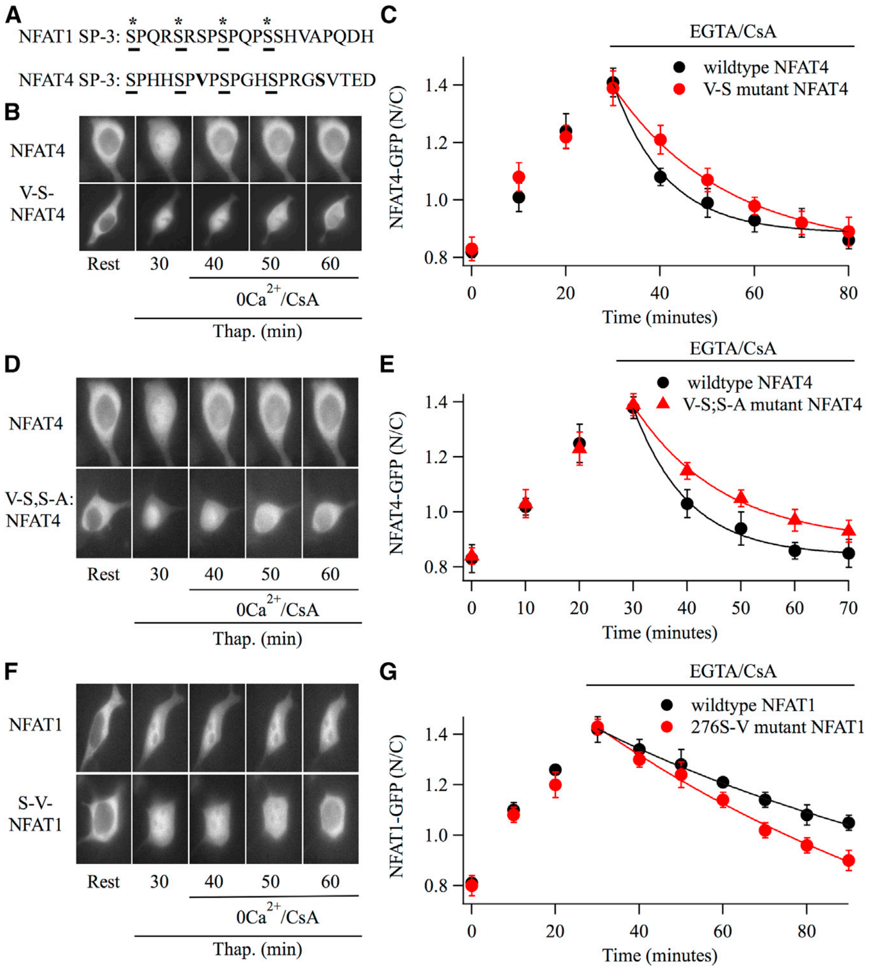


**Figure 7. A Valine to Serine Point Mutation in the SP-3 Motif of NFAT4 Decreases Nuclear Export Rate**

(A) The upper panel compares the sequence of the SP-3 region between NFAT1 and NFAT4. Asterisks denote phosphorylated serine residues in NFAT1 (S270, 274, 278, 282). Conserved serines are underlined. Residues in bold were mutated.

(B) Images compare nuclear dynamics of NFAT4-GFP and V-S-NFAT4-GFP, following CRAC channel activation.

(C) The graph compares nuclear import and export between V-S-NFAT4-GFP and wild-type NFAT4-GFP. Each point is the average (mean ± SEM) of six cells.

(D) Images compare nuclear efflux of wild-type NFAT4-GFP with the double mutant (V-S, S-A-NFAT4-GFP).

(E) Mean data ± SEM from eight cells for each condition are compared.

(F) Images compare nuclear dynamics of wild-type NFAT1-GFP with the S276V-NFAT1-GFP mutant.

(G) Aggregate data (mean ± SEM) from 11 NFAT1-GFP and 14 S276V-NFAT1-GFP cells are compared.

In panels (C), (E), and (G), thapsigargin was applied immediately after obtaining resting images.

the SP-3 domain of NFAT4 was removed and replaced with the corresponding motif from NFAT1 (henceforth referred to as (SP-3NFAT1)-NFAT4)). Although (SP-3NFAT1)-NFAT4-GFP migrated into the nucleus with similar kinetics to NFAT4-GFP after stimulation (Figure 6C), nuclear export of the chimera was considerably slower than for NFAT4-GFP (Figure 6C; mean data are shown on the right; time constants for NFAT4-GFP and (SP-3NFAT1)-NFAT4-GFP were 9.9 ± 2.1 min and 57.4 ± 7.8 min, respectively). These results were confirmed in a gel shift assay, which showed (SP-3NFAT1)-NFAT4-GFP was rephosphorylated more slowly than NFAT4-GFP (Figure 1D). Significantly more (SP-3NFAT1)-NFAT4-GFP accumulated in the nucleus in EGTA-loaded cells after stimulation with thapsigargin (Figure 6D), compared with NFAT4-GFP (Figures 2 and 6D) under similar conditions. Nuclear levels of (SP-3NFAT1)-NFAT4-GFP also increased more than NFAT4-GFP after exposure to thapsigargin in cells expressing either PV-NLS (Figure 6E) or PV-NES (Figure 6F).

Sequence alignments of the SP-3 region of NFAT1 with NFAT4 are compared in Figure 7A. A detailed analysis of phosphorylation sites within the SP-3 domain of NFAT1 has identified four serine residues that are phosphorylated (Okamura et al., 2000), which are marked with asterisks in Figure 7A. These serines in NFAT1 (S270, S274, S278, S282) are conserved in NFAT4. We therefore focused on additional serines that could underlie the differences in nuclear export kinetics. Serine276 in the SP-3 motif of NFAT1 is a valine in NFAT4. We therefore mutated this valine in NFAT4 to a serine (V-S-NFAT4) to see if nuclear export was altered. Although expression of V-S-NFAT4-GFP migrated into the nucleus at a similar rate to wild-type NFAT4-GFP (Figure 7B), nuclear export was ~2-fold slower (Figure 7B; aggregate data are shown in Figure 7C). The time constant for export for wild-type NFAT4 in paired control recordings was 10.9 ± 0.7 min, and this increased to 21.3 ± 2.3 min when V-S-NFAT4 was expressed instead ($p < 0.01$). Another difference is that alanine286 in NFAT1 is a serine in NFAT4. We therefore mutated this serine to alanine in the V-S-NFAT4 mutant. The double mutant V-S/S-A-NFAT4-GFP migrated out of the nucleus with a time course that was not significantly different from V-S-NFAT4-GFP (Figures 7D and 7E), suggesting alanine 286 plays little role in export kinetics.

We made the corresponding mutation in NFAT1, converting serine 276 to a valine (S276V-NFAT1). Compared with wild-type NFAT1-GFP, S276V-NFAT1-GFP moved out of the nucleus slightly faster (~1.2-fold; Figures 7F and 7G), but this was not significant ($p = 0.1$).

## DISCUSSION

Simultaneous expression of multiple protein isoforms is essential for integrated and co-ordinated cellular responses, yet much remains unknown about how isoforms that share the same spatial

domain are differentially activated. We have found that two widely expressed isoforms of the vertebrate transcription factor NFAT, NFAT1 and NFAT4, which are both cytosolic proteins and stimulated by the same $Ca^{2+}$ messenger, require distinct sub-cellular $Ca^{2+}$ signals for activity This requirement for different patterns of cytoplasmic $Ca^{2+}$ enables a physiological agonist to selectively recruit NFAT1 at low stimulus intensities and activate both isoforms as receptor occupancy increases.

NFAT1 is directly coupled to CRAC channels, in that it is selectively activated by $Ca^{2+}$ microdomains near the open channels that extend only a few nanometers below the plasma membrane (Kar et al., 2011, 2012b; Somasundaram et al., 2014). Buffering global cytoplasmic $Ca^{2+}$ with either EGTA or PV-NES or nuclear $Ca^{2+}$ with PV-NLS had no effect on the rate and extent of NFAT1 accumulation within the nucleus. $Ca^{2+}$ release from stores or $Ca^{2+}$ influx through other plasma membrane ion channels (Kar et al., 2011) also fail to activate NFAT1, reinforcing the view of a tête a tête between Orai1 and NFAT1. By contrast, NFAT4 is more promiscuous in its $Ca^{2+}$ requirements. Although $Ca^{2+}$ microdomains near Orai1 channels kick-start NFAT4 activation, a rise in nuclear $Ca^{2+}$ is required to maintain the transcription factor with the nucleus. NFAT4 activation therefore allows for a contribution from multiple sources of $Ca^{2+}$ that convergently elevate global, and thereby nuclear, $Ca^{2+}$.

Our chimera studies ascribed a major role to the SP-3 motif in regulating the kinetics of nuclear export. This motif is phosphorylated by DYRKs (Gwack et al., 2006), and we found that the DYRK kinase inhibitor harmine decelerated NFAT4 export, although to a lesser extent than seen when the isoform was engineered to express the SP-3 domain from NFAT1. Sequence alignment revealed that S276 in NFAT1 is a valine in NFAT4. The V-S-NFAT4 mutant exited the nucleus ~2-fold more slowly than wild-type NFAT4. However, V-S-NFAT4 was still exported about three times more quickly than the (SP-3NFAT1)NFAT4 construct, suggesting other residues within the SP-3 motif make major contributions. Apart from V276, there are eight additional non-conserved amino acid differences between NFAT1 and NFAT4 within the SP-3 motif, and it is likely that several amino acid changes account for the overall difference in kinetics.

The difference in NFAT isoform nuclear export kinetics has important functional consequences. The slow export of NFAT1 enables the protein to activate gene expression even at low-frequency $Ca^{2+}$ spikes, since it will remain within the nucleus long after the $Ca^{2+}$ signal has terminated. By contrast, NFAT4 would be effective only when the $Ca^{2+}$ spike periodicity is shorter (less than the time-constant of export of ~8 min) or when the $Ca^{2+}$ rise is sustained. A simple model that simulates the nuclear dynamics of NFAT1 and NFAT4 is shown in Figure S7. Remarkably, acceleration of only the nuclear export rate ($k_4$) of NFAT4 relative to NFAT1 leads to marked differences in the nuclear levels of each transcription factor. Previous work in T cells showed that different transcription factors responded to different periodicities of the $Ca^{2+}$ signal (Dolmetsch et al., 1998). Our study further suggests that isoforms of the same transcription factor have different $Ca^{2+}$ periodicity requirements.

Finally, the selective dependence of the two NFAT isoforms on distinct sub-cellular $Ca^{2+}$ signatures is of relevance to information processing in immune cells. The tight functional coupling between $Ca^{2+}$ microdomains and NFAT1 activation imparts both selectivity and high fidelity to CRAC channel-transcription coupling (Kar et al., 2012b). Furthermore, by operating locally, this private line of communication is able to trigger gene expression at low concentrations of agonist that recruit a portion of the total CRAC channel pool. By contrast, NFAT4 has a dual dependency on both CRAC channels and a nuclear $Ca^{2+}$ rise. This latter requirement filters out weak stimuli from activating NFAT4-driven transcription since mobilization of a small fraction of the CRAC channel pool and the lack of a prolonged nuclear $Ca^{2+}$ rise conspire to prevent NFAT4 accumulation in the nucleus.

More generally, our results show how closely related protein isoforms can be differentially and sequentially activated by the same agonist in a manner dictated by sub-cellular $Ca^{2+}$ gradients.

## EXPERIMENTAL PROCEDURES

### Cells

HEK293 cells were purchased from ATCC (via the UK supplier LGC) and were cultured at 37°C with 5% $CO_2$ in DMEM supplemented with 10% fetal bovine serum and 1% penicillin/streptomycin, as previously described (Kar et al., 2011).

### cDNA Constructs and Transfection

Cells were transfected using lipofectamine, as described (Kar et al., 2011). cDNA for NFAT4-GFP and all parvalbumin constructs were from Addgene, deposited by Dr. Anjana Rao and Dr. Anton Bennett, respectively. NFAT1-GFP was a gift from Dr. Jennings Worley. The (SP-3NFAT1)-NFAT4-GFP chimera was purchased from Mutagenex. All plasmids were used at 1 μg, and experiments commenced 24–48 hr after transfection.

### NFAT Nuclear Migration

NFAT1-GFP, NFAT4-GFP, and NFAT4-cherry levels in the cytosol and nucleus were measured using the IMAGO charge-coupled device camera-based system from TILL Photonics, with a ×100 oil immersion objective (numerical aperture 1.4). Regions of interest of identical size were drawn in the cytosol and nucleus of each cell, and fluorescence was computed. Nuclear localization was confirmed by co-staining with the nuclear dye DAPI (Kar et al., 2011). Unless otherwise indicated, we calculated the nuclear/cytosolic (N/C) ratio of NFAT-GFP/-cherry as a function of stimulus time. We measured NFAT-GFP translocation in one field of view (approximately one to three cells) for up to 80 min per coverslip. A value of n = 15 therefore represents data from five to eight coverslips (at least three cell preparations).

### Cytoplasmic and Nuclear $Ca^{2+}$ Measurements

Cytoplasmic $Ca^{2+}$ measurements were carried out at room temperature using the IMAGO charge-coupled device camera-based system from TILL Photonics (Kar et al., 2012b). Cells, loaded with Fura-2/AM (1 μM), were alternately excited at 356 and 380 nm (20-ms exposures), at 0.5 Hz. Standard external solution contained 145 mM NaCl, 2.8 mM KCl, 2 mM $CaCl_2$, 2 mM $MgCl_2$, 10 mM D-glucose, and 10 mM HEPES (pH 7.4), with NaOH. $Ca^{2+}$-free solution had the following composition: 145 mM NaCl, 2.8 mM KCl, 2 mM $MgCl_2$, 10 mM D-glucose, 10 mM HEPES, and 0.1 mM EGTA (pH 7.4), with NaOH. $Ca^{2+}$ signals are plotted as *R*, which denotes the 356/380 nm ratio.

Simultaneous measurements of thapsigargin-induced changes in cytoplasmic and nuclear $Ca^{2+}$ were performed with fluo-4. Cells were plated onto glass coverslips and either were not transfected or co-transfected with untagged PV-NLS and mcherry (marker for transfection) or PV-NES-DsRed. Cells were loaded with 2 μM fluo4-AM in a standard external solution for 40 min at room temperature. The changes in fluo-4 fluorescence (488 nm) in the cytosolic and nuclear compartments were monitored with a Zeiss LSM 510 Meta confocal microscope.

### EGTA-AM Loading

Cells were loaded with EGTA-AM (20 μM) or solvent (0.1% DMSO) for 45 min in the dark, as described (Kar et al., 2011).

### Western Blot

Total cell lysates (40 μg) were analyzed by SDS-PAGE on either a 10% (Figure 1D) or 12% (Figure 3A) gel. Membranes were blocked with 5% non-fat dry milk in PBS plus 0.1% Tween 20 (PBST) buffer for 1 hr at room temperature. Membranes were washed with PBST three times and then incubated with primary Ab for 24 hr at 4°C. Total ERK2 and GFP antibodies were from Santa Cruz Biotechnology and Cell Signaling, respectively, and used at dilutions of 1:5,000 (ERK2) and 1:2,000 (GFP), respectively. The membranes were then washed with PBST again and incubated with 1:2,500 dilutions of peroxidase-linked anti-rabbit IgG from Santa Cruz Biotechnology for 1 hr at room temperature. After washing with PBST, the bands were detected by an enhanced chemiluminescence (ECL) plus western blotting detection system (Amersham Biosciences). Blots were analyzed by UN-Scan software. Nuclear and cytoplasmic extracts were separated as described (Chang et al., 2006). Calcineurin levels were normalized to total ERK2 or HH3 (Abcam), for normalized cytoplasmic and nuclear levels (Figure 3A).

### Statistical Analysis

Data are presented as the mean ± SEM. Statistical significance was determined by using the Student's t test, except in Figure 5F, where an ANOVA followed by a post hoc Newman-Keuls multiple comparison test was used (*p < 0.05, **p < 0.01, and ***p < 0.001).

## SUPPLEMENTAL INFORMATION

Supplemental Information includes seven figures and can be found with this article online at http://dx.doi.org/10.1016/j.molcel.2015.02.027.

## AUTHOR CONTRIBUTIONS

P.K. and A.B.P. designed the experiments. P.K. carried out the experiments, and P.K. and A.B.P. analyzed the data. A.B.P. wrote the manuscript.

## ACKNOWLEDGMENTS

This work was supported by an MRC program grant to A.B.P. We thank Dr. Yuris Galvanosky for help with nuclear $Ca^{2+}$ measurements and Dr. Gary Mirams for help with the simulations. A.B.P. is on the board of Calcico Therapeutics.

Received: October 22, 2014
Revised: January 21, 2015
Accepted: February 19, 2015
Published: March 26, 2015

## REFERENCES

Al-Daraji, W.I., Grant, K.R., Ryan, K., Saxton, A., and Reynolds, N.J. (2002). Localization of calcineurin/NFAT in human skin and psoriasis and inhibition of calcineurin/NFAT activation in human keratinocytes by cyclosporin A. J. Invest. Dermatol. *118*, 779–788.

Bird, G.S., and Putney, J.W.J., Jr. (2005). Capacitative calcium entry supports calcium oscillations in human embryonic kidney cells. J. Physiol. *562*, 697–706.

Chang, W.C., Nelson, C., and Parekh, A.B. (2006). Ca2+ influx through CRAC channels activates cytosolic phospholipase A2, leukotriene C4 secretion and expression of c-fos through ERK-dependent and independent pathways in mast cells. FASEB J. *20*, 2381–2383.

Di Capite, J., Ng, S.-W., and Parekh, A.B. (2009). Decoding of cytoplasmic Ca(2+) oscillations through the spatial signature drives gene expression. Curr. Biol. *19*, 853–858.

Dolmetsch, R.E., Xu, K., and Lewis, R.S. (1998). Calcium oscillations increase the efficiency and specificity of gene expression. Nature *392*, 933–936.

Feske, S., Gwack, Y., Prakriya, M., Srikanth, S., Puppel, S.H., Tanasa, B., Hogan, P.G., Lewis, R.S., Daly, M., and Rao, A. (2006). A mutation in Orai1 causes immune deficiency by abrogating CRAC channel function. Nature *441*, 179–185.

Göckler, N., Jofre, G., Papadopoulos, C., Soppa, U., Tejedor, F.J., and Becker, W. (2009). Harmine specifically inhibits protein kinase DYRK1A and interferes with neurite formation. FEBS J. *276*, 6324–6337.

Gwack, Y., Sharma, S., Nardone, J., Tanasa, B., Iuga, A., Srikanth, S., Okamura, H., Bolton, D., Feske, S., Hogan, P.G., and Rao, A. (2006). A genome-wide Drosophila RNAi screen identifies DYRK-family kinases as regulators of NFAT. Nature *441*, 646–650.

Hogan, P.G., Chen, L., Nardone, J., and Rao, A. (2003). Transcriptional regulation by calcium, calcineurin, and NFAT. Genes Dev. *17*, 2205–2232.

Hogan, P.G., Lewis, R.S., and Rao, A. (2010). Molecular basis of calcium signaling in lymphocytes: STIM and ORAI. Annu. Rev. Immunol. *28*, 491–533.

Kar, P., Nelson, C., and Parekh, A.B. (2011). Selective activation of the transcription factor NFAT1 by calcium microdomains near Ca2+ release-activated Ca2+ (CRAC) channels. J. Biol. Chem. *286*, 14795–14803.

Kar, P., Bakowski, D., Di Capite, J., Nelson, C., and Parekh, A.B. (2012a). Different agonists recruit different stromal interaction molecule proteins to support cytoplasmic Ca2+ oscillations and gene expression. Proc. Natl. Acad. Sci. USA *109*, 6969–6974.

Kar, P., Nelson, C., and Parekh, A.B. (2012b). CRAC channels drive digital activation and provide analog control and synergy to Ca(2+)-dependent gene regulation. Curr. Biol. *22*, 242–247.

Kar, P., Samanta, K., Kramer, H., Morris, O., Bakowski, D., and Parekh, A.B. (2014). Dynamic assembly of a membrane signaling complex enables selective activation of NFAT by orai1. Curr. Biol. *24*, 1361–1368.

Leite, M.F., Thrower, E.C., Echevarria, W., Koulen, P., Hirata, K., Bennett, A.M., Ehrlich, B.E., and Nathanson, M.H. (2003). Nuclear and cytosolic calcium are regulated independently. Proc. Natl. Acad. Sci. USA *100*, 2975–2980.

Lewis, R.S. (2007). The molecular choreography of a store-operated calcium channel. Nature *446*, 284–287.

Liou, J., Kim, M.L., Heo, W.D., Jones, J.T., Myers, J.W., Ferrell, J.E., Jr., and Meyer, T. (2005). STIM is a Ca2+ sensor essential for Ca2+-store-depletion-triggered Ca2+ influx. Curr. Biol. *15*, 1235–1241.

Mochly-Rosen, D., and Gordon, A.S. (1998). Anchoring proteins for protein kinase C: a means for isozyme selectivity. FASEB J. *12*, 35–42.

Müller, M.R., and Rao, A. (2010). NFAT, immunity and cancer: a transcription factor comes of age. Nat. Rev. Immunol. *10*, 645–656.

Negulescu, P.A., Shastri, N., and Cahalan, M.D. (1994). Intracellular calcium dependence of gene expression in single T lymphocytes. Proc. Natl. Acad. Sci. USA *91*, 2873–2877.

Neher, E. (1998). Vesicle pools and Ca2+ microdomains: new tools for understanding their roles in neurotransmitter release. Neuron *20*, 389–399.

Okamura, H., Aramburu, J., García-Rodríguez, C., Viola, J.P., Raghavan, A., Tahiliani, M., Zhang, X., Qin, J., Hogan, P.G., and Rao, A. (2000). Concerted dephosphorylation of the transcription factor NFAT1 induces a conformational switch that regulates transcriptional activity. Mol. Cell *6*, 539–550.

Oliveira, A.G., Guimarães, E.S., Andrade, L.M., Menezes, G.B., and Fatima Leite, M. (2014). Decoding calcium signaling across the nucleus. Physiology (Bethesda) *29*, 361–368.

Parekh, A.B. (2008). Ca2+ microdomains near plasma membrane Ca2+ channels: impact on cell function. J. Physiol. *586*, 3043–3054.

Parekh, A.B. (2010). Store-operated CRAC channels: function in health and disease. Nat. Rev. Drug Discov. *9*, 399–410.

Parekh, A.B., and Putney, J.W.J., Jr. (2005). Store-operated calcium channels. Physiol. Rev. *85*, 757–810.

Prakriya, M., Feske, S., Gwack, Y., Srikanth, S., Rao, A., and Hogan, P.G. (2006). Orai1 is an essential pore subunit of the CRAC channel. Nature *443*, 230–233.

Pusl, T., Wu, J.J., Zimmerman, T.L., Zhang, L., Ehrlich, B.E., Berchtold, M.W., Hoek, J.B., Karpen, S.J., Nathanson, M.H., and Bennett, A.M. (2002). Epidermal growth factor-mediated activation of the ETS domain transcription factor Elk-1 requires nuclear calcium. J. Biol. Chem. *277*, 27517–27527.

Seifert, A., Allan, L.A., and Clarke, P.R. (2008). DYRK1A phosphorylates caspase 9 at an inhibitory site and is potently inhibited in human cells by harmine. FEBS J. *275*, 6268–6280.

Shibasaki, F., Price, E.R., Milan, D., and McKeon, F. (1996). Role of kinases and the phosphatase calcineurin in the nuclear shuttling of transcription factor NF-AT4. Nature *382*, 370–373.

Somasundaram, A., Shum, A.K., McBride, H.J., Kessler, J.A., Feske, S., Miller, R.J., and Prakriya, M. (2014). Store-operated CRAC channels regulate gene expression and proliferation in neural progenitor cells. J. Neurosci. *34*, 9107–9123.

Vig, M., Beck, A., Billingsley, J.M., Lis, A., Parvez, S., Peinelt, C., Koomoa, D.L., Soboloff, J., Gill, D.L., Fleig, A., et al. (2006). CRACM1 multimers form the ion-selective pore of the CRAC channel. Curr. Biol. *16*, 2073–2079.

Wu, H., Peisley, A., Graef, I.A., and Crabtree, G.R. (2007). NFAT signaling and the invention of vertebrates. Trends Cell Biol. *17*, 251–260.

Yeromin, A.V., Zhang, S.L., Jiang, W., Yu, Y., Safrina, O., and Cahalan, M.D. (2006). Molecular identification of the CRAC channel by altered ion selectivity in a mutant of Orai. Nature *443*, 226–229.

Yissachar, N., Sharar Fischler, T., Cohen, A.A., Reich-Zeliger, S., Russ, D., Shifrut, E., Porat, Z., and Friedman, N. (2013). Dynamic response diversity of NFAT isoforms in individual living cells. Mol. Cell *49*, 322–330.

Zhang, S.L., Yeromin, A.V., Zhang, X.H.-F., Yu, Y., Safrina, O., Penna, A., Roos, J., Stauderman, K.A., and Cahalan, M.D. (2006). Genome-wide RNAi screen of Ca(2+) influx identifies genes that regulate Ca(2+) release-activated Ca(2+) channel activity. Proc. Natl. Acad. Sci. USA *103*, 9357–9362.

Molecular Cell

Supplemental Information

# Distinct Spatial $Ca^{2+}$ Signatures Selectively Activate Different NFAT Transcription Factor Isoforms

Pulak Kar and Anant B. Parekh

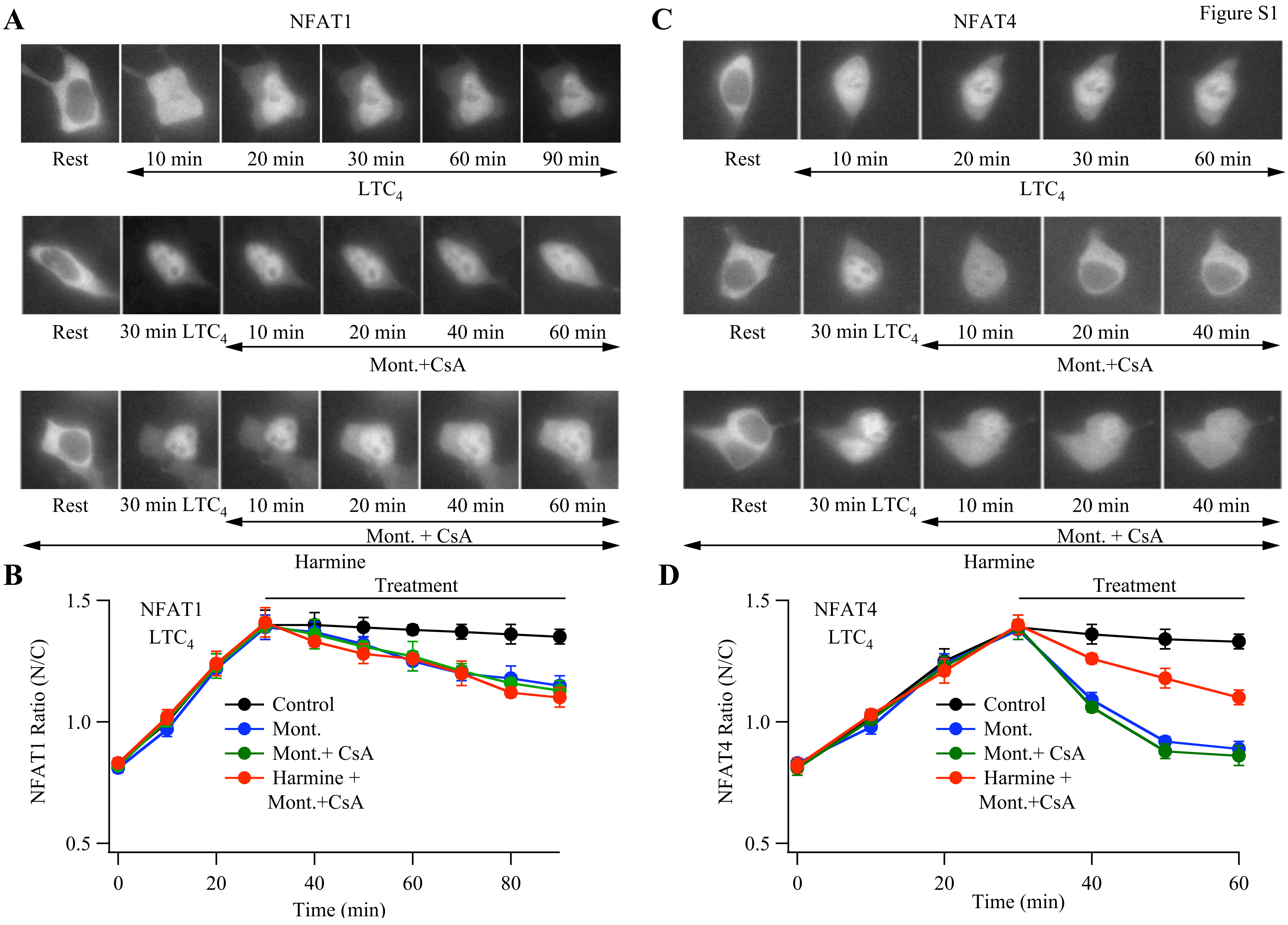
Figure S1
A
NFAT1
Rest
10 min
20 min
30 min
60 min
90 min
LTC4
Rest
30 min LTC4
10 min
20 min
40 min
60 min
Mont.+CsA
Rest
30 min LTC4
10 min
20 min
40 min
60 min
Mont. + CsA
Harmine
C
NFAT4
Rest
10 min
20 min
30 min
60 min
LTC4
Rest
30 min LTC4
10 min
20 min
40 min
Mont.+CsA
Rest
30 min LTC4
10 min
20 min
40 min
Mont. + CsA
Harmine
B
Treatment
NFAT1
LTC4
NFAT1 Ratio (N/C)
1.5
1.0
0.5
0
20
40
60
80
Time (min)
Control
Mont.
Mont.+ CsA
Harmine +
Mont.+CsA
D
Treatment
NFAT4
LTC4
NFAT4 Ratio (N/C)
1.5
1.0
0.5
0
20
40
60
Time (min)
Control
Mont.
Mont.+ CsA
Harmine +
Mont.+CsA

Figure S2

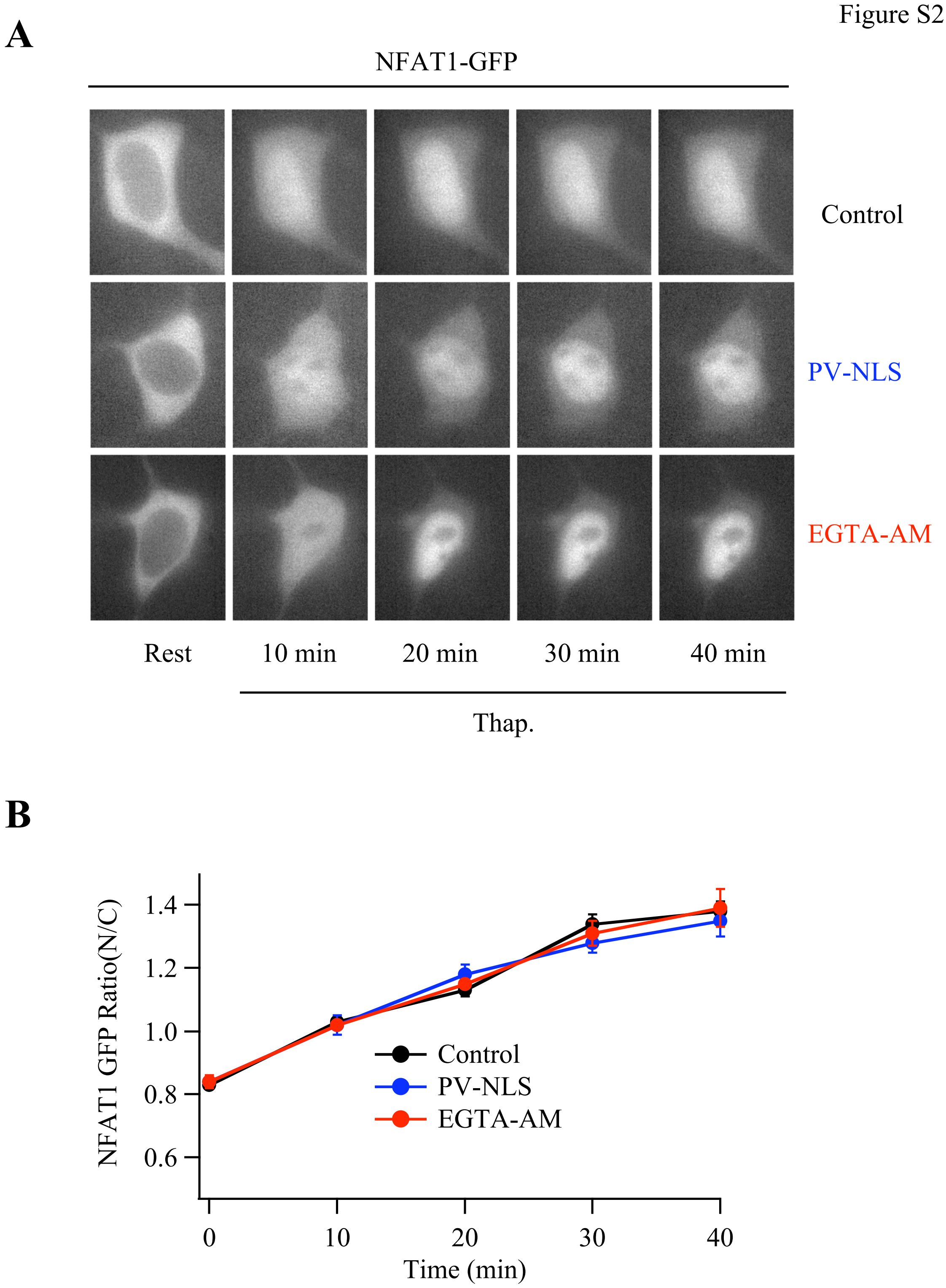

A
NFAT1-GFP
Control
PV-NLS
EGTA-AM
Rest
10 min
20 min
30 min
40 min
Thap.
B
NFAT1 GFP Ratio(N/C)
1.4
1.2
1.0
0.8
0.6
Control
PV-NLS
EGTA-AM
0
10
20
30
40
Time (min)

Figure S3

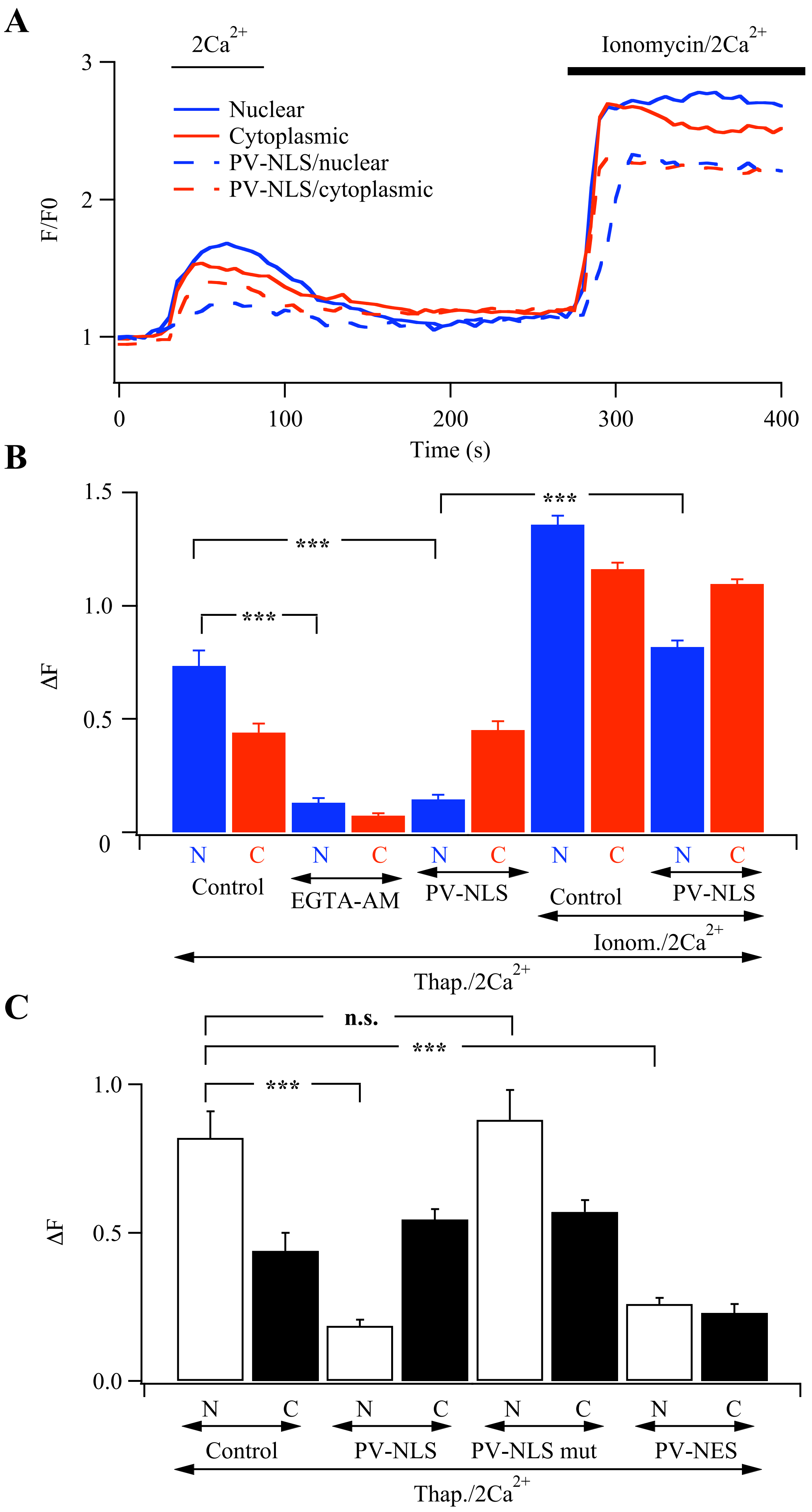
A
2Ca2+
Ionomycin/2Ca2+
Nuclear
Cytoplasmic
PV-NLS/nuclear
PV-NLS/cytoplasmic
F/F0
Time (s)
B
ΔF
***
N C N C N C N C N C
Control
EGTA-AM
PV-NLS
Control
PV-NLS
Ionom./2Ca2+
Thap./2Ca2+
C
n.s.
***
ΔF
N C N C N C N C
Control
PV-NLS
PV-NLS mut
PV-NES
Thap./2Ca2+

Figure S4

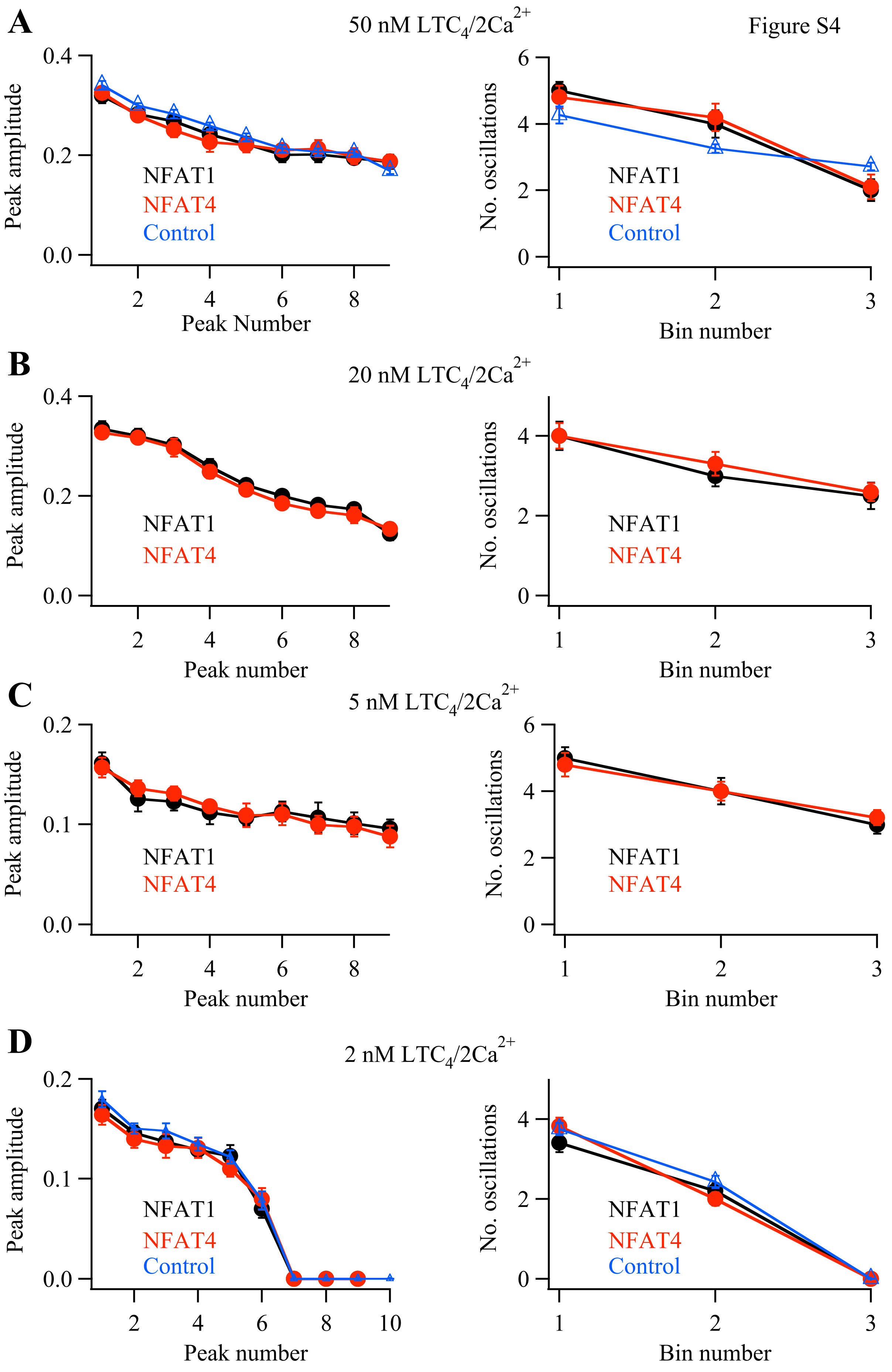
A
50 nM LTC4/2Ca2+
Peak amplitude
0.4
0.2
0.0
2
4
6
8
Peak Number
NFAT1
NFAT4
Control
No. oscillations
6
4
2
0
1
2
3
Bin number
NFAT1
NFAT4
Control
B
20 nM LTC4/2Ca2+
Peak amplitude
0.4
0.2
0.0
2
4
6
8
Peak number
NFAT1
NFAT4
No. oscillations
4
2
0
1
2
3
Bin number
NFAT1
NFAT4
C
5 nM LTC4/2Ca2+
Peak amplitude
0.2
0.1
0.0
2
4
6
8
Peak number
NFAT1
NFAT4
No. oscillations
6
4
2
0
1
2
3
Bin number
NFAT1
NFAT4
D
2 nM LTC4/2Ca2+
Peak amplitude
0.2
0.1
0.0
2
4
6
8
10
Peak number
NFAT1
NFAT4
Control
No. oscillations
4
2
0
1
2
3
Bin number
NFAT1
NFAT4
Control

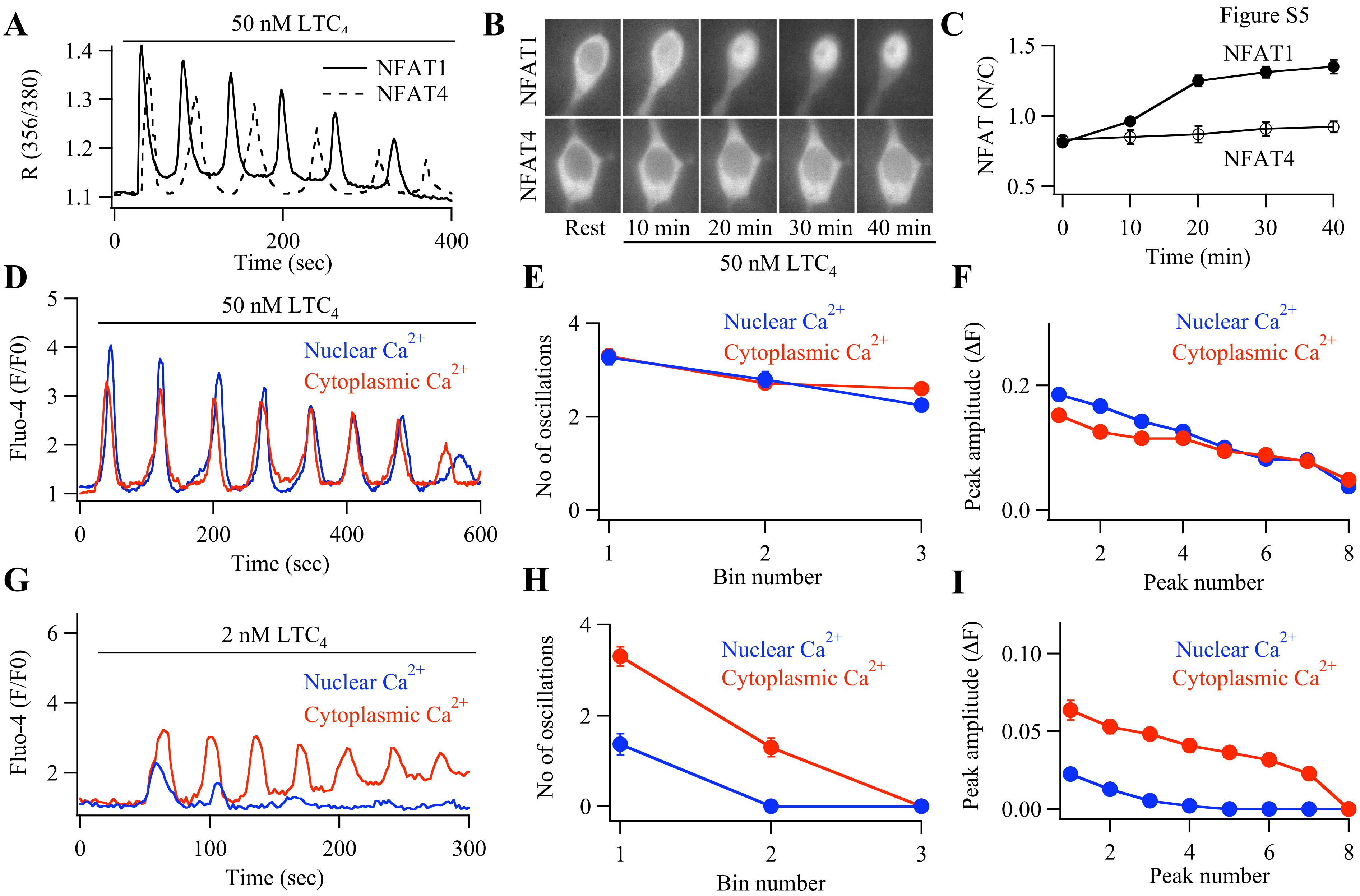

Figure S5
A
50 nM LTC4
NFAT1
NFAT4
R (356/380)
1.4
1.3
1.2
1.1
0
200
400
Time (sec)
B
NFAT1
NFAT4
Rest
10 min
20 min
30 min
40 min
50 nM LTC4
C
NFAT1
NFAT4
NFAT (N/C)
1.5
1.0
0.5
0
10
20
30
40
Time (min)
D
50 nM LTC4
Nuclear Ca2+
Cytoplasmic Ca2+
Fluo-4 (F/F0)
0
200
400
600
Time (sec)
E
Nuclear Ca2+
Cytoplasmic Ca2+
No of oscillations
Bin number
F
Nuclear Ca2+
Cytoplasmic Ca2+
Peak amplitude (ΔF)
0.2
0.0
Peak number
G
2 nM LTC4
Nuclear Ca2+
Cytoplasmic Ca2+
Fluo-4 (F/F0)
0
100
200
300
Time (sec)
H
Nuclear Ca2+
Cytoplasmic Ca2+
No of oscillations
Bin number
I
Nuclear Ca2+
Cytoplasmic Ca2+
Peak amplitude (ΔF)
0.10
0.05
0.00
Peak number

Figure S6

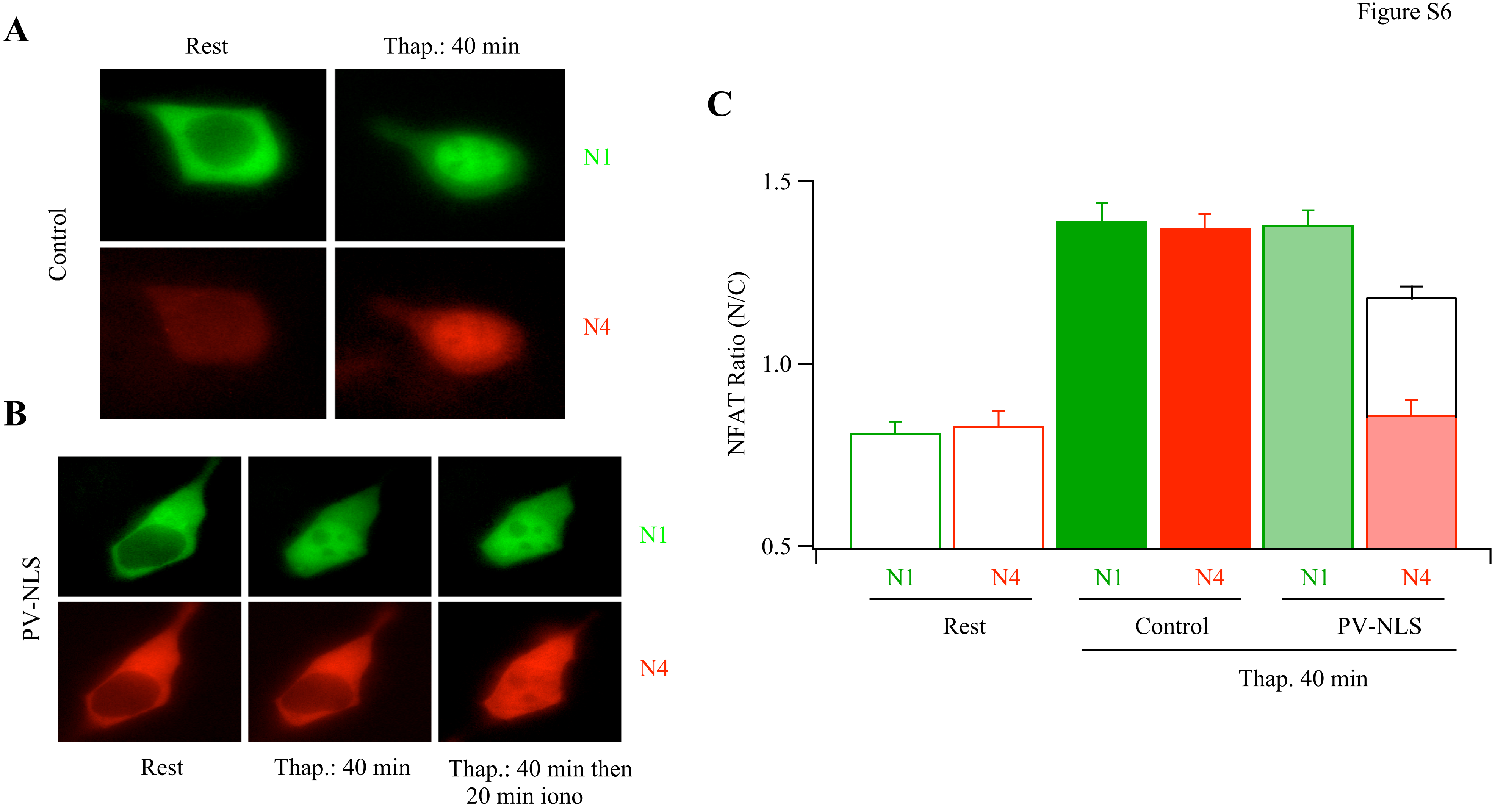

A
Rest
Thap.: 40 min
Control
N1
N4
B
PV-NLS
N1
N4
Rest
Thap.: 40 min
Thap.: 40 min then
20 min iono
C
NFAT Ratio (N/C)
1.5
1.0
0.5
N1
N4
N1
N4
N1
N4
Rest
Control
PV-NLS
Thap. 40 min

**A**

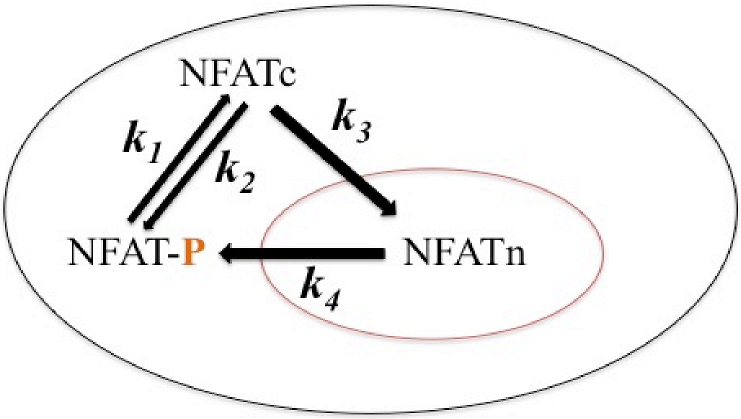


Cytoplasmic phosphorylated NFAT (NFAT-**P**) = X
Cytoplasmic dephosphorylated NFAT (NFATc) = Y
Nuclear NFAT (NFATn) = 1-X-Y

$$dx/dt = -k_1*X + k_2*Y + k_4(1\text{-}X\text{-}Y)$$

$$dy/dt = k_1*X-(k_2+k_3)Y$$

**B**

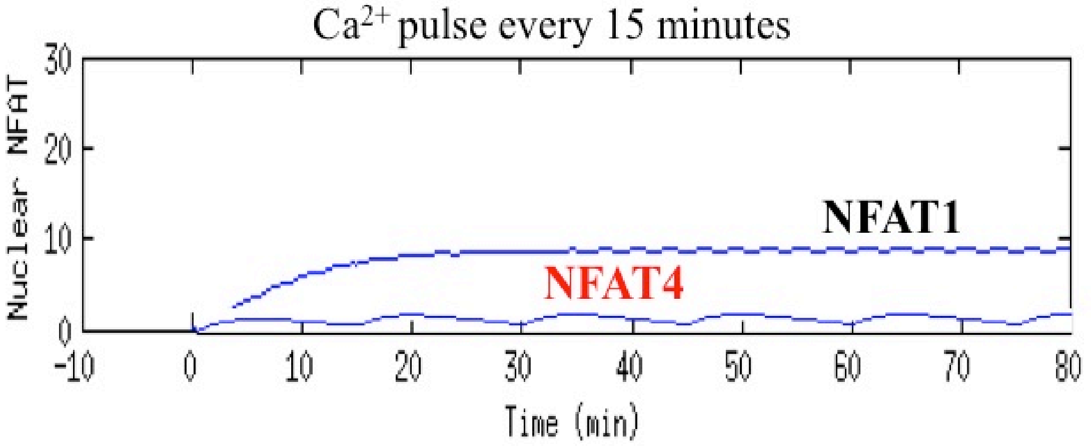


**C**

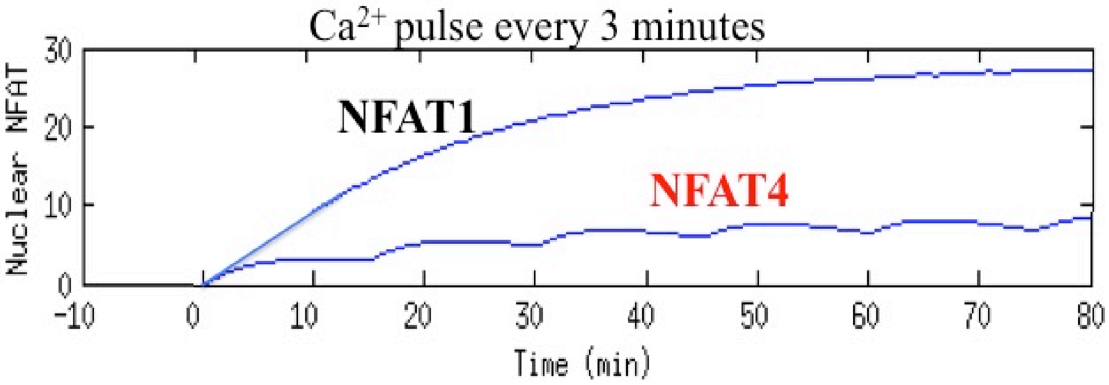


**D**

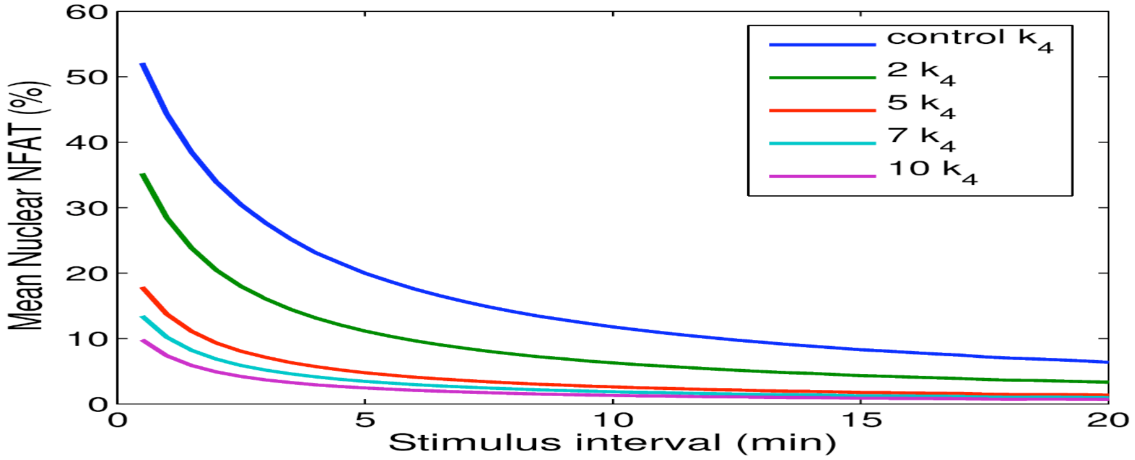

**Figure S1. NFAT4 export from the nucleus is faster than that of NFAT1 following cysLT1 receptor stimulation, related to Figure 1**. (A) Images compare NFAT1 nuclear dynamics following stimulation with 120 nM $LTC_4$. Upper panel shows NFAT1-GFP migration after exposure to agonist for the times indicated. Middle panel depicts NFAT1-GFP export from the nucleus following nuclear accumulation induced by stimulation with $LTC_4$ for 30 minutes. Thereafter, agonist was removed and cells exposed to montelukast (abbreviated to Mont.; 1 μM) and cyclosporine A (CsA; 1 μM). Images show NFAT1-GFP distribution at the times indicated. Lower panel, as in middle panel but now harmine was present too (5 μM pre-incubation for 10 minutes followed by continuous exposure). (B) Aggregate data for the conditions shown are compared. Each point is the average of between 11 and 19 cells. All cells were stimulated with $LTC_4$ shortly after resting images had been obtained. Control denotes the continuous presence of $LTC_4$, Mont. refers to addition of montelukast, Mont. + CsA represents montelukast and cyclosporine A and harmine + Mont.+CsA denotes the presence of all 3 inhibitors. (C) Images as in panel A but now NFAT4-GFP was expressed instead. (D) Aggregate data for NFAT4 for the various conditions are compared. Treatment was as described in panel B for NFAT1. Data are presented as mean±SEM.

**Figure S2. Nuclear accumulation of NFAT1-GFP is unaffected by cytoplasmic $Ca^{2+}$ buffering, related to Figure 3.** (A) NFAT1-GFP translocation to the nucleus in response to thapsigargin stimulation is compared between a control cell, one expressing PV-NLS and one loaded with EGTA. (B) Mean data from several independent experiments are compared. Each point is the mean of between 6 and 13 cells. Error bars denote SEM.

**Figure S3. Simultaneous measurements of nuclear and cytoplasmic $Ca^{2+}$ signals, related to Figure 3.** Nuclear and

cytoplasmic $Ca^{2+}$ was measured with a confocal microscope using fluo-4. (A) Stores were depleted with thapsigargin in $Ca^{2+}$-free solution and 2 mM $Ca^{2+}$ was readmitted, as indicated. Nuclear and cytoplasmic $Ca^{2+}$ levels were measured in wild type cells and in cells expressing PV-NLS. PV-NLS/nuclear indicates nuclear $Ca^{2+}$ in cells expressing PV-NLS. Ionomycin (2 μM) was applied after cytoplasmic $Ca^{2+}$ had returned to basal levels. (B) Mean data for the conditions shown are presented as ΔF, denoting the $Ca^{2+}$ response after stimulation minus the basal level. Each bar is the mean of between 6 and 13 cells. N and C denote nuclear and cytoplasmic $Ca^{2+}$, respectively. (C) Mean data for the conditions shown are compared. PV-NLS mut denotes the PV-NLS mutant that cannot bind $Ca^{2+}$. Error bars denote SEM. *** denotes $p<0.001$ and n.s. not significant.

**Figure S4. Amplitude and frequency of cytoplasmic $Ca^{2+}$ oscillations are maintained over a range of agonist concentrations in cells expressing cysLT1 receptors and NFAT proteins, related to Figure 4.** (A) The amplitude of each oscillation and the number of oscillations per 200 seconds bin are compared following stimulation with 50 nM $LTC_4$. Control denotes cells expressing cysLT1 receptors alone, NFAT1 represents receptors and NFAT1 whereas NFAT4 denotes receptors and NFAT4. (B) Oscillatory responses are compared between cells expressing cysLT1 receptors and NFAT1 with cells expressing cysLT1 receptors and NFAT4 challenged with 20 nM $LTC_4$. (C) Oscillatory responses are compared as in panel B but now 5 nM $LTC_4$ was used. (D) Responses elicited by 2 nM $LTC_4$ are shown under the indicated conditions. Each point represents mean±SEM of 27-36 cells.

**Figure S5. A nuclear $Ca^{2+}$ rise is required for NFAT4 accumulation in the nucleus following physiological levels of stimulation, related to Figure 4.** (A) A low dose of $LTC_4$ evokes

several $Ca^{2+}$ oscillations of similar amplitude and frequency in cells expressing PV-NLS (untagged) and either NFAT1-GFP (solid trace) or NFAT4-GFP (dotted trace). (B) Images compare nuclear accumulation of NFAT1- or NFAT4-GFP following stimulation with $LTC_4$ in cells expressing PV-NLS. (C) Mean data ±SEM from several cells expressing PV-NLS are compared. Each point is the mean of between 14 and 21 cells. (D) Cytoplasmic and nuclear $Ca^{2+}$ are compared for a cell stimulated with 50 nM $LTC_4$. (E) The number of oscillations per 200 seconds bin are presented. (F) The amplitude of each oscillation in the nuclear and cytoplasmic compartments are plotted against oscillation number. Error bars are contained within the symbols. Each graph is the average of 11-14 cells from 2 independent experiments. (G-I) As in panels D-F, but 2 nM $LTC_4$ was the stimulus. Each graph depicts mean data ±SEM from 13-21 cells from 3 independent experiments. In panels D-I, PV-NLS was not expressed.

**Figure S6. PV-NLS suppresses NFAT4 nuclear accumulation but not that of NFAT1 in cells co-expressing both transcription factors, related to Figure 5.** (A) A control cell expressing NFAT1-GFP and NFAT4-cherry was stimulated with thapsigargin and nuclear accumulation is shown after 40 minutes stimulation. (B) Only NFAT1-GFP accumulates in the nucleus after stimulation with thapsigargin in a cell expressing PV-NLS. After 40 minutes stimulation with thapsigargin, the cell was then challenged with 2 μM ionomycin, which led to NFAT4-cherry movement into the nucleus. (C) Mean data from several independent experiments are compared. N1 and N4 denote NFAT1 and NFAT4, respectively. The open bar above N4/PV-NLS shows the subsequent movement after stimulation with ionomycin for 20 minutes. Error bars denote SEM.

**Figure S7. Simulation of NFAT1 and NFAT4 nuclear dynamics following CRAC channel activation, related to Figures 1, 5**

**and 6.** (A) The simplified cartoon depicts NFAT movement into and out of the nucleus and shows the differential equations that were used to simulate NFAT nuclear dynamics. The individual rates for NFAT1 were quantified in Kar et al., 2011. Rates for NFAT4 were obtained from Figure 1. The only difference between NFAT1 and NFAT4 was the nuclear export rate, $k_4$. (B) The graph compares simulated movement of NFAT1 and NFAT4 following low frequency $Ca^{2+}$ pulse of 30 seconds (applied every 15 minutes). A 30 seconds pulse was used to approximate the duration of a $Ca^{2+}$ oscillation in response to cysLT1 receptor activation. (C) In this simulation, $Ca^{2+}$ pulses were applied every 3 minutes. (D) The simulation shows how a change in only the nuclear export rate, $k_4$, profoundly affects NFAT nuclear accumulation. Control $k_4$ corresponds to NFAT1. Increasing export rate, by increasing k4, leads to an exponential increase in NFAT export. 7k4 represents NFAT4, as measured in our experiments. In the simulation, $Ca^{2+}$ pulses of 30 seconds duration were applied, but at different intervals (ranging from once every 30 seconds to once every 20 minutes). The simulations were done with Dr Gary Mirams from the Department of Computer Science at Oxford.
